# ROS inhibits microtubule dynamics and cell growth heterogeneity during Arabidopsis sepal morphogenesis

**DOI:** 10.1016/j.isci.2026.116426

**Published:** 2026-06-23

**Authors:** Isabella Burda, Fridtjof Brauns, Aaron Shipman, Emily Shapland, Lilan Hong, Adrienne H.K. Roeder

**Affiliations:** 1 Genetics, Genomics, and Development Graduate Program, Cornell University, Ithaca, NY 14850, USA; 2 Weill Institute for Cell and Molecular Biology, Cornell University, Ithaca, NY 14850, USA; 3 School of Integrative Plant Science, Section of Plant Biology, Cornell University, Ithaca, NY 14850, USA; 4 Max Planck Institute for the Physics of Complex Systems, Nöthnitzer Straße 38, 01187 Dresden, Germany; 5 Max Planck Institute of Molecular Cell Biology and Genetics, Pfotenhauerstraße 108, 01307 Dresden, Germany; 6 Center for Systems Biology Dresden, Pfotenhauerstraße 108, 01307 Dresden, Germany

**Keywords:** Biological sciences

## Abstract

Developing organs grow to reproducible sizes and shapes, yet the growth of their constituent cells can be highly heterogeneous and fluctuating. During wild-type *Arabidopsis thaliana* sepal development, fluctuations in cell growth average because the fluctuations are not strongly correlated spatially or temporally, and so the sepals grow to uniform sizes and shapes. In contrast, the sepals of the *ftsh4-5* mutant develop to variable sizes and shapes. *FtsH4* encodes a mitochondrial i-AAA protease. Reactive oxygen species (ROS) accumulate in *ftsh4-5* mutants, and lowering ROS levels rescues the sepal size and shape variability. Here, we find that elevated ROS promotes correlated growth fluctuations and causes cortical microtubules to become more “crisscrossed” and stable. The growth rates of the cells with crisscrossed microtubules are lower and more correlated in time, which impairs spatiotemporal averaging of growth. This suggests that ROS affects microtubule dynamics and cell growth fluctuations, which are necessary for robust morphogenesis.

## Highlights

• High ROS inhibits uncorrelated fluctuations in cell growth in Arabidopsis sepals
• High ROS promotes “crisscrossed” microtubules, which are more stable
• Cells with crisscrossed microtubules also have correlated growth fluctuations

## Graphical abstract

**Figure:**
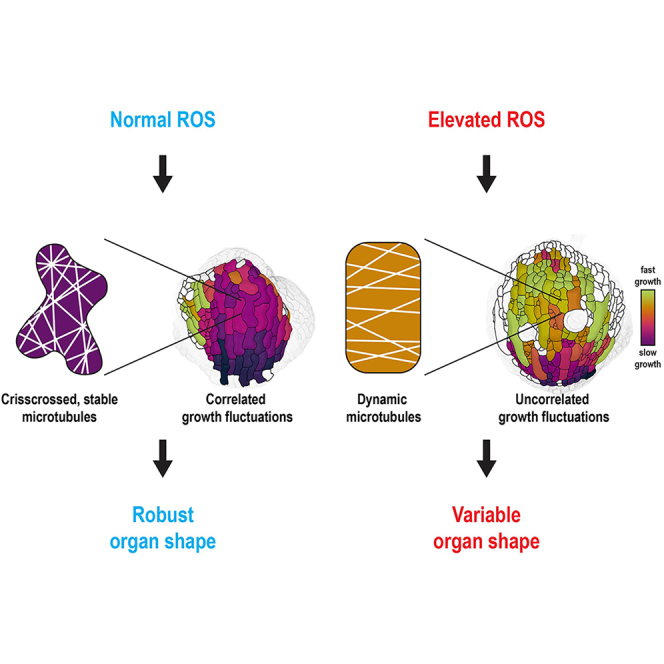

## Introduction

Organ size and shape are important for function.^1^ For example, organs need to scale to be the correct size for the organism or have specialized shapes for certain tasks. Arabidopsis flowers have four sepals—the leaf-like organs on the outside of the flower—which are uniform in size and shape.^2^^,^^3^ Uniform sepal size and shape allow flower buds to remain closed while the inner floral organs are developing, protecting them from the environment. Sepal size and shape are consistent, reproducible outcomes of development despite fluctuations in cell growth and division,^2^^,^^4^^,^^5^ meaning that development is robust or not easily influenced by noise.

Cell growth rates fluctuate, and growth directions can be heterogeneous, strongly varying between neighboring cells and over time.^2^^,^^5^^,^^6^^,^^7^^,^^8^ Although it seems counterintuitive for fluctuating, heterogeneous growth to lead to a reproducible final outcome, sometimes heterogeneous cell behavior can be more effective than homogenous cell behavior for achieving robust morphogenesis. For example, during the closing of the *Xenopus laevis* neural tube, spatially random cell constriction occurs, and modeling suggests this is more effective at folding the tissue than simultaneous constriction.^9^

As an illustrative example, consider data analysis, where one applies spatial and temporal (in short, spatiotemporal) averaging to smooth or denoise data. By the law of large numbers, when many independent (or weakly correlated) random fluctuations are averaged, their mean converges to the expected value. As a result, small-scale variability tends to cancel out, allowing large-scale trends to emerge clearly. This principle helps explain robustness in organ size and shape during morphogenesis.^1^^,^^2^^,^^4^^,^^10^ The final shape of an organ arises from the cumulative growth and division of its many individual cells. Because individual cell growth rates are variable, one might expect large variability in the final morphology. However, if these growth fluctuations are uncorrelated (and especially if they are anticorrelated) across space and time, their effects will average out across the tissue. Their aggregate growth will then converge toward the expected large-scale growth pattern. Importantly, spatiotemporal averaging does not determine the organ’s size or shape. Those are set by overarching growth programs (for example, the basipetal growth gradient in sepals). Rather, averaging ensures that stochastic deviations at the cellular level do not propagate into large-scale morphological variability. If every cell perfectly followed the prescribed growth pattern, averaging would be unnecessary. But in real biological systems, cell behavior is noisy and heterogeneous. Because many cells contribute over extended space and time, these fluctuations statistically cancel, allowing the organ to reliably converge to its specified size and shape despite underlying cellular variability.^1^^,^^2^^,^^4^^,^^10^

This spatiotemporal averaging of heterogeneity has been shown to occur during sepal development. In wild-type sepals, epidermal cell growth rates fluctuate spatially between neighboring cells and temporally during development. Since fluctuations in growth do not have strong spatial or temporal correlations, the cumulative growth over a longer time interval is similar for all cells.^2^^,^^4^ This spatiotemporal averaging of heterogeneity can lead to uniform final sepal size and shape. Averaging of heterogeneity is also a generalizable principle; for example, noise in transcript levels has also been shown to average over time and space in a tissue.^11^

Spatiotemporal averaging and robust development are disrupted in the *ftsh4-5* mutant,^2^^,^^4^ which is a null mutant for the mitochondrial i-AAA protease filamentation temperature sensitive H4 (FtsH4).^12^ Fluctuations in growth rate in *ftsh4-5* are temporally and spatially more correlated than in wild type, meaning that cells grow faster or slower than the target growth rate for too long in spatial patches. As a result, some patches of cells over-grow and some patches of cells under-grow, leading to asymmetric patterns of cumulative growth, and variable size and shape between sepals.^4^ Thus, correlated heterogeneity is detrimental to robust organ development as it prevents averaging from reducing fluctuations. However, there are also examples of increased growth heterogeneity causing sepal shape variability, such as in the *vip3* mutant.^13^ While reducing spatial or temporal correlations in growth fluctuations should facilitate averaging, increasing the magnitude of these fluctuations should inhibit averaging. It is unclear whether fluctuations in growth have a purpose in morphogenesis, besides those from differentiating cell types, or if growth fluctuations are unavoidable noise.

Mutants for *ftsh4* also have abnormal mitochondria morphology with few cristae^14^ and accumulate aggregates of proteins largely composed of mitochondrial small heat shock proteins.^2^^,^^15^ These defects in the *ftsh4* mitochondria suggested that reactive oxygen species (ROS) might be involved, as these are a normal byproduct of aerobic respiration that are toxic at high levels, and have also been co-opted as signaling molecules.^16^ ROS are elevated in *ftsh4-5* mutants, which is linked to the severity of the serrated leaf phenotype.^14^ Overexpression of *CATALASE2*, which breaks down hydrogen peroxide, decreases ROS levels and rescues *ftsh4-5* sepal variability phenotype.^2^ Since elevated ROS causes loss of robust sepal development, we hypothesized that ROS may promote correlated growth fluctuations, which hamper spatiotemporal averaging and robust sepal development in *ftsh4-5*.

Multiple factors have been found to contribute to fluctuations in growth. In the development of the Arabidopsis sepal epidermis, some of these fluctuations derive from changes in growth rate as cells differentiate into specialized cell types^7^ or from decreased growth of cells neighboring the differentiating cells, which buffers the fast growth.^7^^,^^17^ In the *vip3* mutant, increased noise in gene expression has been associated with increased spatial fluctuations in growth, which leads to organ shape variability,^13^ yet it is not known whether stochastic gene expression in wild type also drives a reduction in correlations in growth. In the shoot apical meristem, mutants in a pair of actin genes have fewer spatial fluctuations in cell growth.^18^ Spatial fluctuations are also decreased when microtubule severing is impaired in *katanin1* mutant meristems.^8^ The mutant *mor1-1*, which also affects microtubule dynamics, slightly decreases spatial correlations in subcellular growth direction near abnormal cell shapes.^19^ Although some factors contributing to correlations in growth rate are known, it is unclear how these factors fit into pathways to create uncorrelated growth fluctuations.

Plant cell growth occurs when the cell wall is deformed by turgor pressure; therefore, the mechanical properties of the cell wall could affect growth heterogeneity. Cellulose microfibrils are polysaccharide chains that are a component of the cell wall,^20^ and their layout confers anisotropic mechanical properties to the cell wall.^21^ The cell wall will be stiff in the direction parallel to cellulose microfibrils; as a result, turgor pressure deforms the wall perpendicular to the cellulose microfibrils.^21^ Cellulose synthases move along cortical microtubules,^22^ and the direction of new cellulose microfibrils added to the cell wall should mirror the cortical microtubule arrangement. Therefore, cell growth direction is predicted to be perpendicular to microtubules as well as the cellulose microfibrils.^21^

There is also a relationship between microtubules and correlations in growth. As stated above, *katanin1* mutants have decreased spatial fluctuations in growth rate.^8^ Depolymerizing microtubules enhances the increased spatial fluctuations in growth rate in the *vip3* mutant, suggesting that microtubules may partially compensate for the effect of *vip3* on growth fluctuations.^13^ Cortical microtubules respond to differences in growth rate between cells by aligning parallel to the tension generated in cells neighboring a rapidly growing cell. This causes the neighboring cells to decrease their growth rate.^17^ Thus, mechanical tension orients microtubules that guide cell growth through cellulose microfibril arrangement. In turn, growth reshapes mechanical tension, creating a continuous mechanical feedback loop. Modeling suggests that the strength of mechanical feedback to growth fluctuations can also affect the spatial correlations in growth fluctuations.^23^ A small amount of mechanical feedback in cells neighboring a growth fluctuation can dampen the spatial correlation in the fluctuation, whereas a large enough mechanical response can overcorrect for the fluctuation.^23^

Here, we investigate the effect of ROS levels on the spatiotemporal averaging of growth rate fluctuations. We also test whether ROS affects microtubule dynamics. We find that elevated ROS levels inhibit uncorrelated growth fluctuations and increase microtubule stability. The cells with decreased microtubule dynamics also display temporally correlated growth, although depolymerizing the microtubules is insufficient to restore normal growth fluctuations. Our results show that ROS homeostasis, microtubule dynamics, and low correlations in cell growth fluctuations contribute to robust morphogenesis.

## Results

### ROS inhibits cell growth heterogeneity

**Figure 1:**
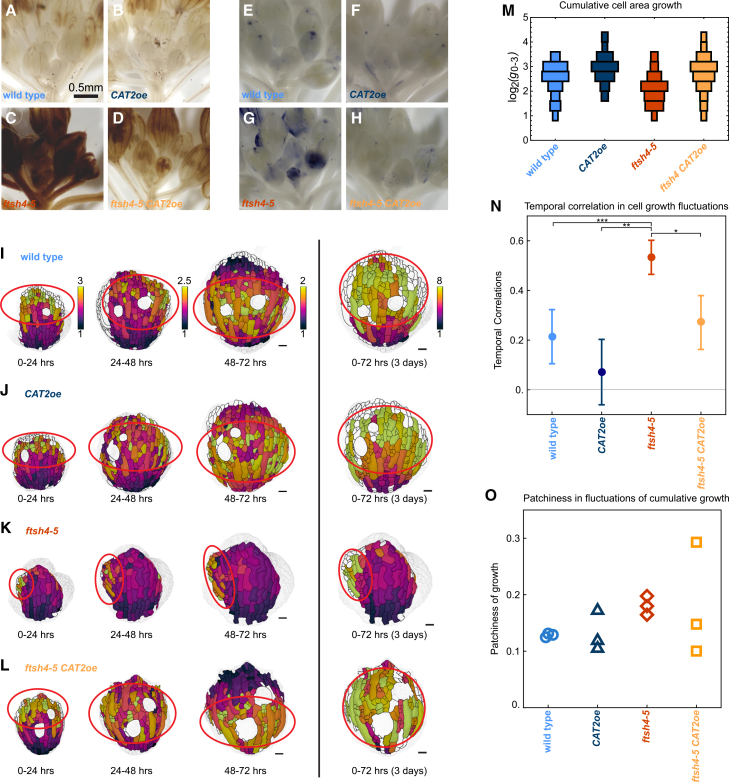
ROS inhibits growth heterogeneity (A–D) Inflorescences stained for hydrogen peroxide. Scale bars are 0.5 mm. Representative images from *n* = 3 biological replicates. *CAT2oe* and *ftsh4-5 CAT2oe* are heterozygous for individual insertions of *CAT2oe*, and from the same plants that were live imaged. (A) Wild type and (B) *CAT2oe* have similar levels of hydrogen peroxide, (C) *ftsh4-5* has elevated levels of hydrogen peroxide, and (D) *ftsh4-5 CAT2* has a partially rescued level. (E–H) Inflorescences stained for superoxide. (E) Wild type and (F) *CAT2oe* have similar levels of superoxide, (G) *ftsh4-5* has elevated levels of superoxide, and (H) *ftsh4-5 CAT2* has a partially rescued level. (I–L) Cell area growth represented as a ratio of the cell area at the later time point divided by the cell area at the earlier time point, projected on the later time points over 24-h intervals and cumulative over 3 days. (I) Wild-type and (J) *CAT2oe* cell growth follows a basipetal (base to tip) growth pattern with local heterogeneity over 24 h intervals, and the heterogeneity accumulates evenly over 3 days. (K) *ftsh4-5* cell growth follows a basipetal growth pattern, but with growth asymmetrically localized within the organ-scale pattern, and it accumulates into patches of more or less cumulative growth over 3 days. (L) *ftsh4-5 CAT2* cell growth is rescued and is similar to wild type over 24 h intervals and 3 days. Scale bars are 20 μm. Representative images from *n* = 3 sepals. (M) Histograms of area growth rate in all four genotypes (*n* = 3 sepals) with cell growth calculated as a ratio of final area to initial area. (N) Plot of the average value of temporal correlation in growth rates for all cells imaged in a sepal (for *n* = 3 sepals; data represent means with bars showing 0.05–0.95 quantile calculated by bootstrapping analysis). The fluctuations in growth rate around the average growth for each time point and replicate are calculated, and then the temporal correlation in fluctuations over subsequent 24 h intervals is calculated. To calculate growth fluctuations, the growth rate along the proximal-distal axis is fit with a third-order polynomial, which is then considered the average growth. The difference between actual growth rates and the average growth rate is the fluctuations in growth. For more details on how fluctuations were calculated, see Methods and Burda et al. 2024. (O) Patchiness of growth over 3-day is calculated by the standard deviation of the fluctuations after averaging the fluctuation over the immediate neighboring cells. For all calculations, *n* = 3 sepals, which include 157 cells for wild type, 113 cells for *CAT2oe*, 169 cells for *ftsh4-5*, and 141 cells for *ftsh4-5 CAT2oe*. ∗*p* < 0.1, ∗*p* < 0.01, and ∗∗∗*p* < 0.01 which obtained using z-test for correlation coefficients. Related to Figure S1.

The loss of robustness mutant, *ftsh4-5*, has elevated ROS, and it was previously shown that the overexpression of *CATALASE2* rescues both ROS levels and the size and shape variability in mature sepals.^2^ It was also previously shown that temporally correlated fluctuations in cell growth during *ftsh4-5* sepal development inhibited spatiotemporal averaging, leading to variable organ size and shape.^4^ Here, we tested whether decreasing ROS also rescues the temporally correlated growth fluctuations in *ftsh4-5*. To do this, we first created *ftsh4-5*, overexpression of *CATALASE2* (*p35S*:*:CAT2*, hereafter referred to as *CAT2oe*), *ftsh4-5 CAT2oe*, and wild-type plants that also had fluorescent membrane markers and microtubule markers. The *CAT2oe* and *ftsh4-5 CAT2oe* plants were heterozygous for individual insertions of the transgene. Independent insertions of *CAT2oe* had differing expression levels of *CATALASE2* (Figure S1A), and genotyping confirmed the transgene was segregating in the offspring. To check how *CAT2oe* affected ROS levels, inflorescences were stained for hydrogen peroxide (Figures 1A–1D; S1B) and superoxide (Figures 1E–1H and S1C), which are two species of ROS. ROS levels in wild type are low in young flower buds (Figures 1A and 1E), later appearing at the tip of sepals in older flowers and progressing proximally down sepals as flowers continue to develop^2^ (Figures S1B and S1C). The spatial localization of ROS appears similar in *CAT2oe* as in wild type. *CAT2oe* has slightly lower levels of ROS, suggesting that it has only a marginal effect when ROS are already at wild type levels (Figures 1B and 1F, S1B, and S1C). In *ftsh4-5*, ROS are elevated in flowers of all developmental stages (Hong et al., 2016) (Figures 1C and 1G, S1B, and S1C). However, in *ftsh4-5 CAT2oe*, ROS are lowered in young flowers compared to *ftsh4-5* (Figures 1D and 1H), and the localization appears similar to the wild-type localization (Figures S1B and S1C). Thus, low ROS accumulation correlates with genotypes that were previously shown to have sepal size and shape robustness.^2^ Our results confirm rescue of the *ftsh4-5* high ROS phenotype by promoting ROS breakdown, as shown previously (Hong et al., 2016).

To test whether ROS levels affect correlations in growth fluctuations and spatiotemporal averaging, we time-lapse live-imaged sepal development of these plants, which express fluorescent markers for the plasma membrane (*pUBQ10*:*:mCherry-RCI2A*) and microtubules (*pUBQ10*:*:GFP-MBD*) (Figures 1I–1L). Sepals have an organ-scale basipetal growth gradient,^2^^,^^4^^,^^7^^,^^17^ meaning that faster growth starts at the tip and moves to the base. To measure local growth heterogeneity, we model cell growth rates along the proximal-distal axis and then quantify the fluctuations from the average growth. This “subtracts” the changes in growth rate due to the basipetal gradient, revealing the local heterogeneity. Then we calculate the correlation in fluctuations of each cell between time intervals (Figure 1N; as described previously in Burda et al., 2024).

If local fluctuations in growth rate are not strongly correlated in space or time, then growth averages over space and time.^2^^,^^4^ In the raw growth rates, which also reflect the basipetal growth gradient, spatiotemporal averaging is visible as the 3-day cumulative growth patterns appear smooth and symmetrical compared to the 24-h growth patterns (Figures 1I–1L). Wild type has low temporal correlations in growth rate fluctuations, and so over the 3-day imaging series, fluctuations average and the cumulative cell growth rates are similar between cells (Figures 1I, 1N, S1D, and S1E), indicating spatiotemporal averaging of heterogeneity.^2^^,^^4^ *CAT2oe* also has low temporal correlations in growth fluctuations, which average in the 3-day cumulative cell growth rates (Figures 1J, 1N, S1F, and S1G). Growth fluctuations are temporally correlated in *ftsh4-5*, with regions of cells that grow slower than average over all 24-h time intervals, and regions of cells that grow at a rate similar to wild type and fluctuate, resulting in 3-day cumulative cell growth rates that appear asymmetric and patchy (Figures 1K, 1N, S1H, and S1I), indicating a loss of spatiotemporal averaging.^4^ In *ftsh4-5 CAT2oe*, temporal correlations in growth fluctuations are lowered to levels similar to wild type, and the cumulative 3-day growth is averaged like wild type (Figures 1L, 1N, and S1J, and S1K), consistent with the restoration of robust sepal morphology. Additionally, the cumulative growth rate is decreased in *ftsh4-5*, likely due to the contribution of slow-growing patches of cells with temporally correlated growth, and partially rescued in *ftsh4-5 CAT2oe* although the difference does not reach significance (Figure 1M). Together, this indicates that decreasing ROS with *CAT2oe* restores growth heterogeneity in *ftsh4-5* development, as well as the previously shown restoration of mature sepal morphology.^2^

Increased temporal correlations in growth heterogeneity lead to cumulative growth that is unevenly distributed in the organ, with patches of overgrowth and undergrowth.^4^ Patchiness is quantified by averaging the fluctuation in 3-day cumulative growth of each cell with its immediate neighboring cells and then calculating the variability in these averaged fluctuations (Figure 1O; as previously described in Burda et al., 2024). A lower score indicates more evenly distributed cumulative growth, whereas a higher score indicates more unevenly distributed or patchy cumulative growth. Patchiness of the 3-day growth is partially rescued in *ftsh4-5 CAT2oe*, but the differences between groups do not reach significance (Figure 1O). The difference in significance between these results and previous results^4^ is likely due to the slightly slower growth of the microtubule marker line, so that the *ftsh4-5* patchy growth is not as pronounced in the same time interval. The variability in the rescue comes from natural variation in the severity of the *ftsh4-5* phenotype and likely from differences in the expression of *CAT2* in different transgenic lines. Together, these data suggest that the disruption of robust organ size and shape from elevated ROS,^2^ occurs through increasing temporal correlations in growth, which are known to inhibit spatiotemporal averaging.^4^

### Increasing ROS leads to a crisscrossed microtubule arrangement

**Figure 2:**
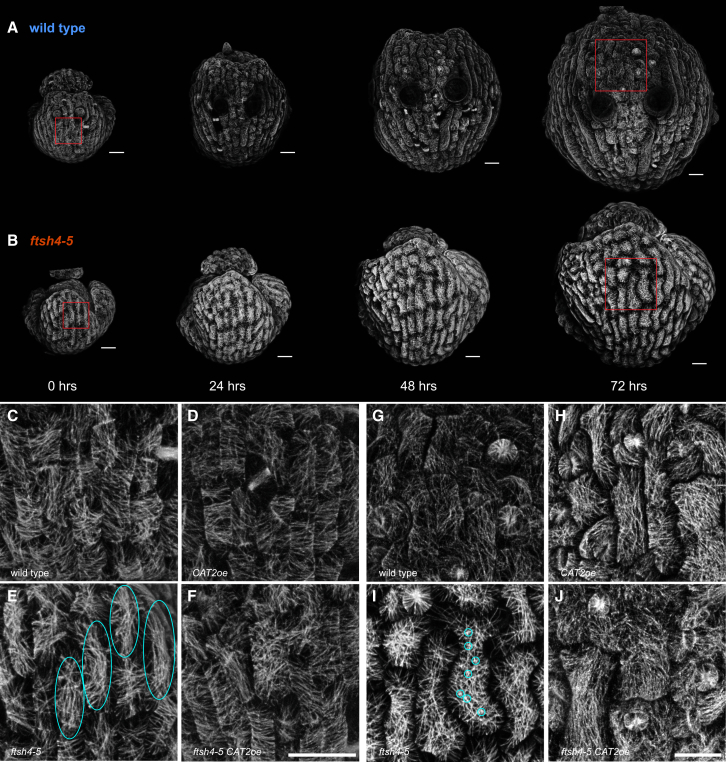
: Microtubule arrangement is different in *ftsh4-5* and rescued in *ftsh4-5 CAT2oe* (A and B) Microtubule signal from the live time-lapse imaging of sepal development. Representative replicates of (A) wild type, and (B) *ftsh4-5* from *n* = 3 sepals. Scale bars are 20 μm. Red boxes indicate the location of the zoomed-in panels. (C–F) Microtubule arrangement on the first day of imaging (developmental stage 5) for (C) wild type, (D) *CAT2oe*, (E) *ftsh4-5*, (F) *ftsh4-5 CAT2oe*. Panels are zoomed in to show a few cells from the time-lapse imaging. Representative images from *n* = 3 replicates. Scale bars are 20 μm. Cyan ovals in (E) point out cells with longitudinal microtubules. (G–J) Microtubule arrangement at the last day of imaging (developmental stage 7) for (G) wild type, (H) *CAT2oe*, (I) *ftsh4-5*, (J) *ftsh4-5 CAT2oe*. Panels are magnified to show a few cells from the time-lapse imaging. Representative images from *n* = 3 sepals. Scale bars are 20 μm. Cyan circles in (I) point out the “star-shapes” in one cell from many microtubules crossing over each other. Additional replicates in Figure S2.

Next, we investigated whether changes in cell growth heterogeneity correlated with differences in microtubule arrangement. Since the microtubule marker was imaged simultaneously with the membrane marker, we examined the microtubule arrangement of individual cells at each time point of the live imaging series from Figures 1 and S1 (Figures 2A, 2B, and S2). At the beginning of the time series (corresponding to floral stage 5), microtubules in wild type appear isotropic (meaning oriented in all directions) or slightly anisotropic in the transverse direction (parallel to the short axis of the cell) (Figure 2C). *CAT2oe* microtubules appear similar to wild type (Figure 2D). However, microtubules in *ftsh4-5* often appear longitudinal (parallel to the long axis of the cell) (Figure 2E). This is rescued in *ftsh4-5 CAT2oe*, which has microtubules arranged similarly to wild type (Figure 2F).

At the end of the time series (fourth time point, corresponding to floral stage 7), microtubules in wild type are isotropic (Figure 2H), as are microtubules in *CAT2oe* sepals (Figure 2I). The microtubule arrangement in *ftsh4-5* appears different in some patches of cells (Figure 2J). Although the microtubules are isotropic, there are many locations where the microtubule signal crosses over from many angles, so that its signal looks star-shaped. We describe this microtubule arrangement as “crisscrossed;” it is an extreme degree of isotropy. The microtubule arrangement is rescued in *ftsh4-5 CAT2oe* (Figure 2K) and usually shows levels of isotropy like those in wild type. Therefore, microtubule arrangements are similar between genotypes with normal growth heterogeneity at the same developmental stage, whereas *ftsh4-5* microtubules have distinctly different microtubule arrangements at each developmental stage.

To assess whether the fluorescent microtubule marker transgene affects the microtubule arrangement, we also created independent insertions of a fluorescently tagged tubulin (*p35S*:*:RFP-TUB6*; Figure S3). We find that *ftsh4-5 p35S*:*:RFP-TUB6* still has patches of cells with abnormal arrangements of microtubules; however, these patches have longitudinal microtubules at the developmental stages that *ftsh4-5 pUBQ10*:*:GF-MBD* has crisscrossed microtubules. This is likely a minor difference since many microtubules are longitudinal to create a crisscrossed arrangement, and cells with crisscrossed microtubules have predominantly longitudinal microtubules earlier in development. A similar difference between microtubule reporter lines has been previously reported; after compression, plants expressing *GFP-MBD* have crisscrossed, bundled microtubules, whereas plants expressing *GFP-TUA6* have some crisscrossed microtubules and some depolymerized, diffuse signal.^24^ Microtubules returned to the pre-compression organization after recovery in both microtubule marker lines.^24^ Since the *ftsh4-5 p35S*:*:RFP-TUB6* sepals still have the variable *ftsh4-5* sepal size and shape phenotype, the microtubule marker transgenes have minor effects on microtubule arrangement, which do not affect organ growth. For clarity, we will still refer to the *ftsh4-5* arrangement as crisscrossed; however, the microtubules that are longitudinal in the crisscrossed arrangement may have more biological importance.

**Figure 3:**
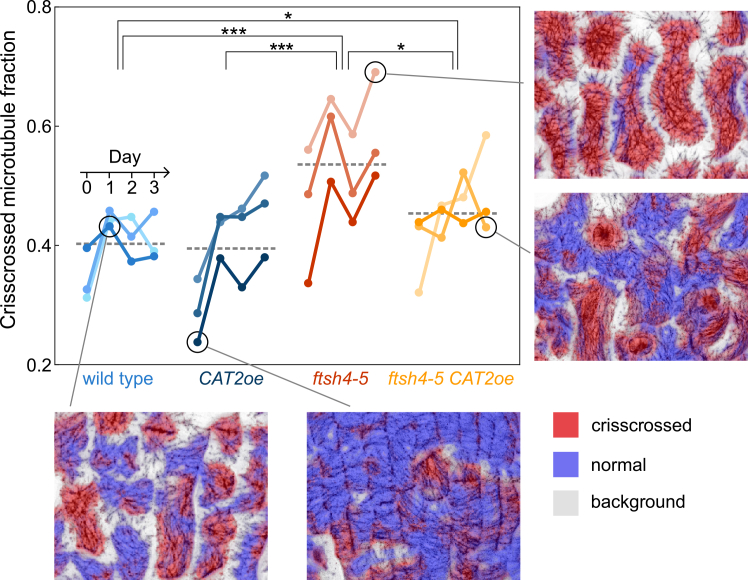
Microtubules in *ftsh4-5* are more crisscrossed An image classifier was trained to classify microtubules as organized or crisscrossed on a subcellular scale using the microtubule signal from the live imaging. The “crisscrossed microtubule fraction” was calculated as the proportion of pixels classified as crisscrossed (represented in red) vs. normally organized (represented in blue). Pixels that are white are background and were excluded from the analysis because they often contain a distorted signal from microtubules on the anticlinal walls. ∗*p* < 0.05 and ∗∗∗*p* < 0.001 from a Student's t-test between genotype pairs using data from all replicates and days.

To quantify differences in microtubule arrangement between genotypes at all time points, we used the pixel classifier implemented in ilastik^25^ to classify the microtubule signal as organized (normal) or crisscrossed (Figure 3). The classifier was trained on manually labeled ground truth data for salient instances of crisscrossed vs. normal microtubules. An additional label was used to mark the spaces between cells and the background with very low or no microtubule signal. Applying the trained classifier yields images where probabilities for the labels are assigned to each pixel (see color overlay in Figure 3). Instances of crisscrossed microtubules (red label) can occur in all genotypes but are most prevalent in *ftsh4-5* (Figure 3, top right) and least abundant in *CAT2oe* (Figure 3, bottom right). Quantifying the tissue area fraction where microtubules appear crisscrossed in each image, confirms that crisscrossed microtubules were more prevalent in *ftsh4-5* at all time points compared to other genotypes. *CAT2o*e had the lowest prevalence of crisscrossed microtubules, and *ftsh4-5 CAT2oe* is a partial rescue with a crisscrossing fraction between wild type and *ftsh4-5* (Figure 3). In wild type, *CAT2oe*, and *ftsh4-5 CAT2oe* cells, microtubules are typically oriented transversally. However, there are also regions with isotropic microtubules that can appear crisscrossed and are classified as such. Therefore, this classification supports that *ftsh4-5* has an abnormal, crisscrossed microtubule arrangement that is partially rescued by the overexpression of *CAT2*.

**Figure 4:**
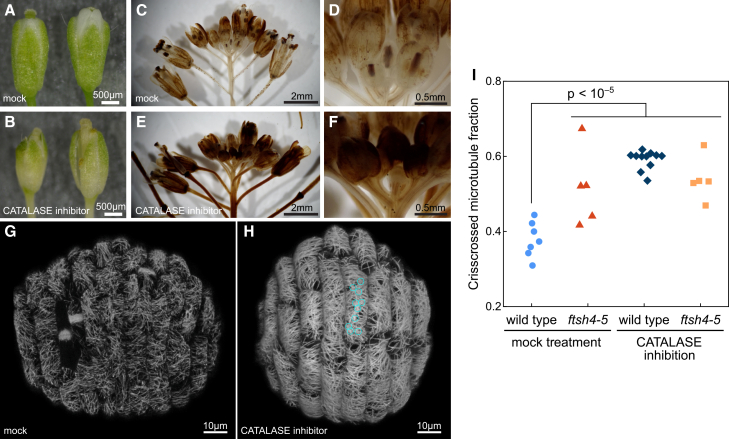
ROS is sufficient to lead to crisscrossed microtubules (A and B) Flowers from mock-treated (A) and 3-AT-treated (B) inflorescences. 3-AT treatment causes variability in organ size and shape. Representative images from *n* = 5 flowers. (C–F) Inflorescences are stained for hydrogen peroxide with DAB. (C) Whole mock-treated inflorescences have hydrogen peroxide in the sepals of flowers in later development and mature flowers, as seen previously. (D) Magnification of the youngest flowers shows that hydrogen peroxide is low. (E) Whole 3-AT-treated inflorescences have elevated hydrogen peroxide levels throughout, and (F) magnification of the youngest flowers shows that hydrogen peroxide is elevated in the youngest flowers as well. Representative images from *n* = 7 inflorescences. (G and H) Microtubules in (G) mock-treated and (H) CATALASE-inhibitor-treated sepals. Cyan circles point out star shapes from crisscrossed microtubules in one cell. Representative images from *n* = 11 sepals. (I) Plot of the proportion of microtubules classified as crisscrossed (red pixels) or anisotropic (blue pixels) in the mock vs. CATALASE-inhibitor-treated sepals. Representative images show that red pixels are classified as crisscrossed and blue pixels are classified as anisotropic. White pixels were excluded from the analysis because they often contain a distorted signal from microtubules on the anticlinal walls. *p* values were calculated using Student t-tests. Additional replicates in Figures S4 and S5.

To determine whether the elevated ROS in *ftsh4-5* is sufficient to cause crisscrossed microtubules, we elevated ROS using the CATALASE inhibitor 3-AT (3-amino-1,2,4-triazole). Plants with the microtubule marker were treated for 5 days, which leads to flowers with sepals of small or variable size, which is similar to the *ftsh4* variable organ size and shape phenotype (Figures 4A, 4B, S4A, and S4B). As expected, treatment with 3-AT elevated hydrogen peroxide in wild-type plants compared to the mock treatment (Figures 4C–4F, S4E, and S4F). The 3-AT treatment also further elevated ROS levels in *ftsh4-5* and enhanced the variable organ size phenotype compared to the mock treatment (Figures S4C, S4D, S4G, and S4H). The microtubules in mock-treated and 3-AT plants were also imaged after 5 days of treatment (Figures 4G, 4H, and S5). Wild-type plants treated with 3-AT had microtubules that were brighter and crisscrossed (Figures 4H, 4I, and S5B), in a manner that is strikingly similar to the microtubule arrangement in *ftsh4-5*. The microtubules in the mock treatment were isotropic with a slight bias in the transverse direction (Figures 4G, 4I, and S5A), similar to those of the untreated wild type. In *ftsh4-*5, 3-AT treatment also increased the brightness and the amount of crisscrossed microtubules, enhancing the phenotype (Figures S5C and S5D). The increase in brightness and contrast in the microtubule signal appears similar to the change that occurs in *GFP-MBD* meristems that are compressed for 6.5 h, which was attributed to microtubule bundling.^24^ The recapitulation of crisscrossed microtubule arrangement by increasing ROS with 3-AT and the rescue of microtubule arrangement by decreasing ROS with *CAT2oe ftsh4-5* indicates that ROS causes the microtubule phenotype in *ftsh4-5* and is sufficient to cause the crisscrossed microtubules. However, the change in morphology was not obvious before 4 or 5 days of treatment; therefore, the effect on microtubules could be indirect, rather than an immediate response.

### Microtubules are more dynamic in wild type than in *ftsh4-5*

**Figure 5:**
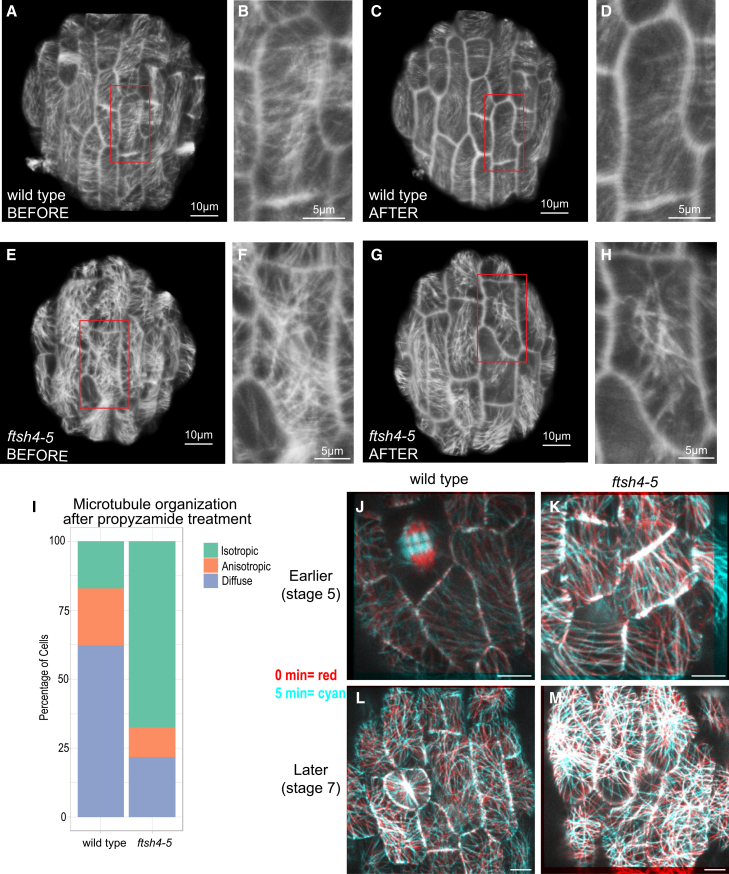
Crisscrossed microtubules in *ftsh4-5* are more stable (A–I) Propyzamide treatment to compare microtubule stability. (A) Wild-type sepal cells before treatment, and the red box marks the location of the magnification in (B). (C) The same wild-type sepal cells after 30 min propyzamide treatment, and the red box marks the location of the magnification in (D). (E) *ftsh4-5* sepal cells before treatment, and the red box marks the location of the magnification in (F). (G) The same *ftsh4-5* sepal cells after 30 min propyzamide treatment, and the red box marks the location of the magnification in (H). (I) The arrangement of microtubules in each cell was scored after propyzamide as anisotropic, isotropic, or depolymerized (which appears as a diffuse signal in the cell). Scores are displayed as a percentage of total cells in a stack bar graph for wild type and *ftsh4-5* for *n* = 53 wildtype cells from 5 sepals and *n* = 46 *ftsh4-5* cells from 5 sepals. (J–M) Microtubules are imaged every 5 min and displayed as a merge of the 0 min time points in red and the 5 min time points in cyan. Separated colors indicate changes in microtubule orientations. Red and Cyan merge into white, which indicates microtubules that are present at both time points. Microtubules are imaged in wild type (J) and *ftsh4-5* (K) at the developmental stage corresponding to the first day of the live imaging time series (stage 5). Representative images of *n* = 3 replicates. Scale bars are 4 μm. Microtubules are imaged in wild type (L) and *ftsh4-5* (M) at the developmental stage corresponding to the last day of the live imaging time series (stage 7). Representative images of *n* = 3 replicates. Scale bars are 4 μm. Additional replicates in Figure S6 and Videos S2, S3, S4, and S5.

Next, we tested whether the different microtubule arrangements in wild type and *ftsh4-5* corresponded to a difference in microtubule dynamics. Microtubules are dynamic in both wild type and *ftsh4-*5 and polymerize quickly after photobleaching (Figure S6A, Video S1). We also tested the stability of microtubules with a propyzamide treatment, which inhibits microtubule polymerization.^26^ Thus, comparing the amount of microtubule depolymerization in a given amount of time allows for comparison of microtubule stability. Propyzamide treatment has been used in many studies to assess microtubule stability. For example, in tobacco cells, the microtubules remaining after a few minutes of propyzamide treatment are in close proximity to each other, meaning they are bundled.^27^ In roots grown in media with a low level of propyzamide, microtubules have decreased rates of both polymerization and depolymerization,^28^ which also indicates remaining microtubules are less dynamic. We imaged 30 min after treatment, when many microtubules have depolymerized, and the remaining microtubules are more stable and less dynamic. This was done in wild-type and *ftsh4-*5 sepals at stage 5–6 (corresponding to the second day of live imaging) (Figures 5A–5H and S6). Microtubules in wild type appeared isotropic before treatment (Figures 5A, 5B, and S6B), and after treatment most but not all cells had only diffuse signal, indicative of depolymerized microtubules (Figures 5C, 5D, and S6C). Microtubules in *ftsh4-5* appeared isotropic before treatment (Figures 5E, 5F, and S6D) and after treatment most cells still had polymerized microtubules remaining (Figures 5G, 5H, and S6E). The reduced depolymerization of microtubules in *ftsh4-5* suggests that *ftsh4-5* cortical microtubules are more stable. Although all cortical microtubules are dynamic, this suggests that microtubules in *ftsh4-5* persist longer before catastrophe or treadmilling. As a control, the microtubules in the preprophase bands depolymerized completely in both genotypes, indicating that this is not a difference in sensitivity to the treatment (Figures S6F–S6I). The organization of the microtubules that remained after treatment also differed between wild type and *ftsh4-5*. In wild type, remaining microtubules were often anisotropic in the transverse direction (Figure 5D) even when microtubules were isotropic before treatment (Figure 5B), whereas the remaining microtubules in *ftsh4-5* were usually isotropic (Figure 5H) and had a similar organization before and after treatment (Figure 5F). When the arrangement of the microtubules was scored per cell after treatment, wild type had more cells with fully depolymerized microtubules, and more cells that had anisotropic microtubules remaining compared to *ftsh4-5* (Figure 5I). These results suggest that there is a difference in microtubule stability and a difference in the orientation of the most stable microtubules between wild type and *ftsh4-5*.

Video S1. Microtubule polymerization rate is similar in wild type and *ftsh4-5*, related to Figure 5 Fluorescently tagged tubulin is bleached in wild type (top row) and *ftsh4-5* (bottom row) sepals and imaged every 10 s after bleaching for 300 s.

To examine microtubule dynamics more directly, we imaged microtubules once every 5 min. In both wild-type and *ftsh4-5* sepals at the stage corresponding to 0 h time point in the live imaging series (stage 5) there were many changes in individual microtubules in each 5 min interval (Figures 5J and 5K, Video S2). At this developmental stage, *ftsh4-5* microtubules are often longitudinal (Figure 2E). In some cells with longitudinal microtubules, the microtubules reorganize to be more crisscrossed for one or a few frames, and then again become longitudinal (Video S2). This suggests that there is not a unidirectional transition from longitudinal to crisscrossed microtubules in *ftsh4-5*. Our analysis suggests that microtubules in *ftsh4-5* and wild type are dynamic at this stage of sepal development, with many microtubules changing within 5 min time intervals.

Video S2. 5 min intervals show microtubule dynamics are similar in wild type and *ftsh4-5* at earlier developmental stage, related to Figure 5 Wild-type (top row) and *ftsh4-5* (bottom row) sepals that correspond to the first time point of live imaging are imaged every 5 min for 70 min total.

In the developmental stage that corresponds to the last day of the live imaging series (stage 7), wild-type microtubules are isotropic and still have many changes over each 5 min interval (Figure 5L and Video S3). In *ftsh4-5*, while there are still changes in microtubules over each 5 min, crisscrossed microtubules persist longer than the other microtubules (Video S3 Figure 5M). This result indicates that crisscrossed microtubules in *ftsh4-5* have increased stability. It is likely that these less dynamic crisscrossed microtubules that persist longer in *ftsh4-5* are the same stable isotropic microtubules that remained in *ftsh4-5* after propyzamide treatment. Also, it should be noted that despite substantial dynamics in individual microtubules, the cell-scale general orientation of microtubules did not change over the 65 min total imaged in either development stage or genotype, suggesting that 24 h time intervals are still informative of average cell-scale microtubule direction.

Video S3. 5 min intervals show crisscrossed microtubules persist longer than other microtubules, related to Figure 5 Wild-type (top row) and *ftsh4-5* (bottom row) sepals that correspond to the last time point of live imaging are imaged every 5 min for 70 min total.

To look at finer timescale microtubule dynamics, we also imaged microtubules every 10 s at the developmental stage 5 (Video S4) and stage 7 (Video S5). Altogether, the results of these experiments suggest that wild-type microtubules continue to be dynamic during sepal development, whereas crisscrossed microtubules in *ftsh4-5* have enhanced stability.

Video S4. 10 s intervals show crisscrossed microtubules persist longer at the earlier developmental stage, related to Figure 5 Wild-type (top row) and *ftsh4-5* (bottom row) sepals that correspond to the first time point of live imaging are imaged every 10 s for 200 s total.

Video S5. 10 s intervals show crisscrossed microtubules persist longer at the later developmental stage, related to Figure 5 Wild-type (top row) and *ftsh4-5* (bottom row) sepals that correspond to the last time point of live imaging are imaged every 10 s for 200 s total.

### Cells with temporally correlated growth have crisscrossed microtubules

**Figure 6:**
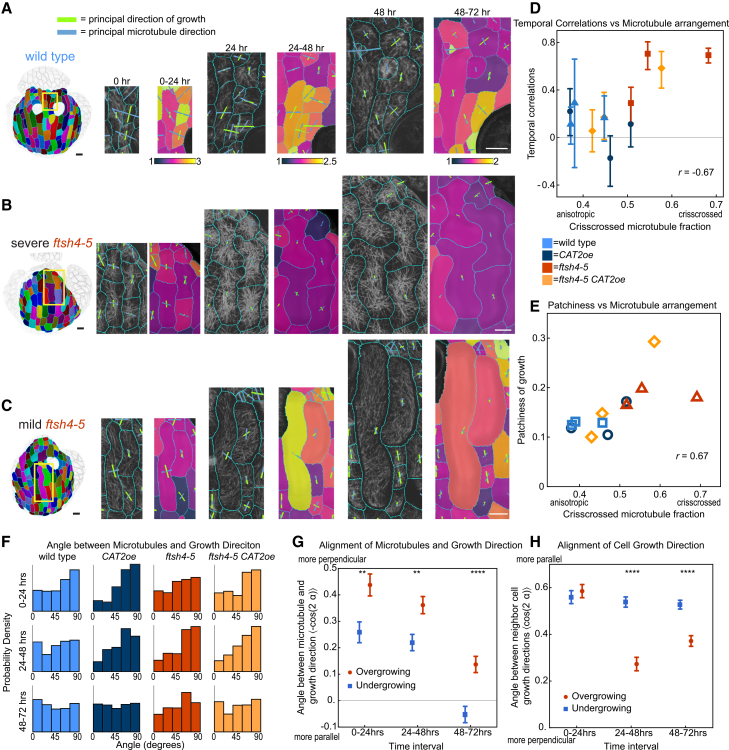
Cells with decreased growth heterogeneity have crisscrossed microtubules, and growth direction is biased toward perpendicular to microtubule direction (A–C) Microtubule direction, growth rate, and growth direction can be obtained from the same live imaging dataset. Scale bars are 10 μm. (A) Wild-type sepal cells with heterogeneous growth rate have transverse or isotropic microtubules. (B) Cells with decreased growth heterogeneity in *ftsh4-5* have microtubules that are longitudinal and then crisscrossed. (C) Cells with heterogeneous growth in *ftsh4-5* have microtubules that look more similar to those in wild type. (D) The temporal correlation strength in growth fluctuations from Figure 1N plotted against the fraction of crisscrossed microtubules from Figure 3 for individual replicates. Data represent the means with bars showing 0.05–0.95 quantiles calculated by bootstrapping analysis. Correlation coefficient 0.67. (E) The score for patchiness in growth fluctuations from Figure 1O plotted against the crisscrossed microtubule fraction from Figure 3 for individual replicates. Correlation coefficient 0.63. (F) Weighted histograms of the angle between microtubule direction per cell and cell principal direction of growth plotted for each genotype and time interval. Histograms are also weighted by microtubule anisotropy and growth direction anisotropy by (max - min)/(max + min) for both values, and using the product of these values as weights. Each genotype has *n* = 3 Sepals, which include 157 cells for wild type, 113 cells for *CAT2oe*, 169 cells for *ftsh4-5*, and 141 cells for *ftsh4-5 CAT2oe*. (G) Plot of average alignment between microtubule direction and growth direction for cells overgrowing relative to their neighbors and undergrowing relative to their neighbors. Data are represented as means with bars showing standard error of the mean (SEM). Data from all genotypes are pooled. Average alignment is measured as ⟨cos(2*α*)⟩. The quantity –cos(2α) is 1 if the microtubule and growth directions are perpendicular (*α* = *π*/2), and -1 if the microtubule and growth directions are parallel (*α* = 0). Note that we chose the sign such that the expected outcome, perpendicular orientation of growth and microtubules, corresponds to a positive alignment measure. (H) Plot of average alignment between neighboring cell growth directions for cells overgrowing relative to their neighbors and undergrowing relative to their neighbors. Data are represented as means with bars showing SEM. Data from all genotypes are pooled. Average alignment is measured as ⟨*cos*(2*α*)⟩. The quantity cos(2α) is 1 if the growth directions of a cell and its neighbors are parallel (*α* = 0), and -1 if if the growth directions of a cell and its neighbors are perpendicular (*α* = *π*/2) for all cells. ∗∗ means *p* value < 0.01 and ∗∗∗∗ means *p* value < 0.0001 which were calculated using student t-tests.

We next tested whether the differences in the arrangement and stability between wild-type microtubules and *ftsh4-5* crisscrossed microtubules correlated with differences in cell growth in our live imaging time series. We tested this because we noticed that both slow-growing cells and cells with crisscrossed microtubules occurred in patches (Figures 6A–6C). While microtubules are generally thought to control cell growth anisotropy, not rate, the pattern in which cellulose is deposited may also affect cell growth rate. For example, the microtubules rapidly reorient from transverse to longitudinal when the hypocotyl is exposed to light.^22^ This rotation is associated with a steep decline in cell growth rates. We compared microtubule arrangements in cells with normal growth in wild type (Figure 6A), cells in slow temporally correlated growth patches in *ftsh4-5* (Figure 6B), and cells in normal growth patches in *ftsh4-5* (Figure 6C). Cells in slow temporally correlated patches in *ftsh4-5* have longitudinal microtubules at earlier time points and crisscrossed microtubules at later time points (Figure 6B). However, microtubules in normal-growth patches in *ftsh4-5* looked similar to wild type (Figure 6C). Since the *ftsh4-5* sepal phenotype is variable with a range of severity, we also looked at the correlation between the average microtubule organization for each sepal and the growth heterogeneity scores (Figures 1N and 1O) for the same sepal. We find that replicates with more crisscrossed microtubules have more temporal correlation in growth fluctuations (Figure 6D) and patchier accumulation of growth (Figure 6E). Therefore, the same *ftsh4-5* cells have correlated growth fluctuations and crisscrossed microtubules.

### Growth direction is biased toward perpendicular to the microtubule direction

Cell walls are expected to deform perpendicular to cellulose microfibril direction,^21^ and therefore it is often assumed that cell growth direction is perpendicular to microtubule direction. However, wild-type sepal cells often have isotropic microtubules despite organ elongation in the proximal-distal direction. We tested whether this relationship could be resolved on the cell scale, since there is variability in growth direction between cells.^2^^,^^4^ We examined the angle between cell-scale microtubule direction and cell growth direction, which is expected to be 90°. The analysis was also weighted by the anisotropy of microtubule direction and growth direction, since it is expected that microtubule anisotropy would cause growth anisotropy. We found that the angle between the principal direction of cell growth over 24 h and the principal microtubule orientation at the start of the time interval is biased toward 90° in many of the genotypes and time intervals (Figure 6F). The 0 to 24h time interval in wild type, *CAT2oe*, and *ftsh4-5 CAT2oe* are the most biased toward 90° (Figure 6F). To further resolve the relationship between microtubules and growth direction, we tested whether the angle was affected by growth rate. We find that the angle between microtubule and growth direction is significantly closer to perpendicular in cells that are overgrowing than in cells that are undergrowing relative to their neighbors (Figure 6G). This result suggests that an increased growth rate may cause microtubules to have a greater influence on cell growth direction. We then hypothesized that growth direction is also influenced by the growth directions of neighboring cells, as cells cannot move past each other due to the cell walls. To test this, we measured the angle between growth directions of neighboring cells and found that cells that are overgrowing relative to their neighbors have growth directions that are less aligned with those of neighboring cells (Figure 6H). This means that surrounding cells can influence growth direction, and that faster growth overcomes some of this influence. Together, these results suggest that cell growth direction is influenced by both microtubule direction in a cell and neighboring cell growth directions. Faster growth causes the growth direction to be influenced more by its own microtubule direction rather than the neighboring cells.

### Depolymerization of microtubules is not sufficient to restore normal cell growth fluctuations

**Figure 7:**
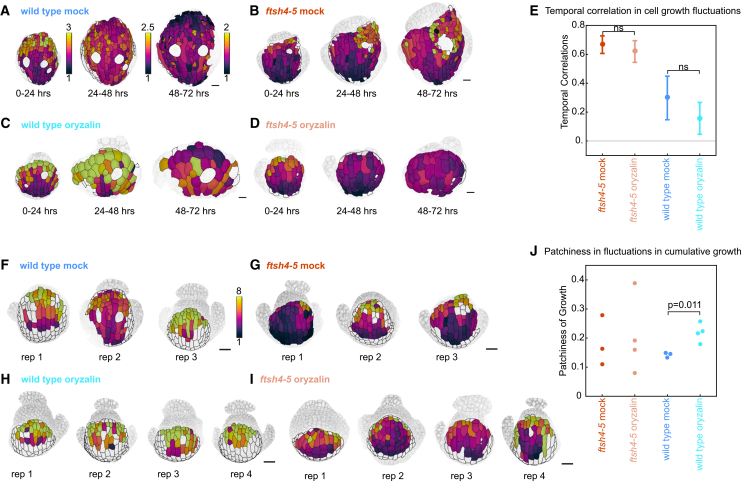
Depolymerizing the microtubules is insufficient to restore growth heterogeneity (A–D) Cell area growth heat maps of sepal development with an oryzalin treatment. Sepals were imaged once every 24 h for 4 days. Area growth is represented as a ratio and projected onto the later time point. Scale bars are 20 μm. Representative time series for (A) wild type mock treatment, (B) *ftsh4-5* mock treatment, (C) wild type oryzalin treatment, (D) *ftsh4-5* oryzalin treatment. *n* = 3 for mock treatments and *n* = 4 for oryzalin treatments to account for fewer cells from decreased division. (E) The fluctuations in growth rate around the average for each time point and replicate are calculated, and then the temporal correlation in fluctuations over subsequent 24 h intervals is calculated. Data are represented as means with bars showing 0.05–0.95 quantiles calculated by bootstrapping analysis. ns means nonsignificant, and differences between all other groups are significant. (F–I) Heat maps of cumulative cell area growth over 3 days for all replicates of (F) wild type mock treatment, (G) *ftsh4-5* mock treatment, (H) wild type oryzalin treatment, and (I) *ftsh4-5* oryzalin treatment. (J) Patchiness of growth over 3 days is calculated by the standard deviation of the fluctuations after averaging the fluctuation over the immediate neighboring cells. Scale bars are 20 μm. For calculations, *n* = 3 sepals for mock treatments and *n* = 4 sepals for oryzalin treatments, which include 126 cells for wild type, 121 cells for *CAT2oe*, 143 cells for *ftsh4-5*, and 162 cells for *ftsh4-5 CAT2oe*. *p* values were obtained using a z-test for correlation coefficients. Additional replicates in Figure S7. Also related to Figure S8.

Since cells with crisscrossed microtubules display increased temporal correlation of growth fluctuations, we tested whether depolymerizing the microtubules could decrease correlations in growth. We time-lapse live-imaged wild type and *ftsh4-5* sepal development once every 24 h for 4 days, as was done for the previous live-imaging series (Figures 7A–7D and S7). We used an oryzalin treatment to depolymerize microtubules, which began one day before the start of imaging, so microtubules were partially depolymerized on the first day of imaging and fully depolymerized on the second day of imaging (Figure S8). As previously reported, oryzalin treatment (Figures 7C, 7D, S7C–S7E, and S7H–S7J) caused the cells to become more isotropic in shape.^29^^,^^30^ This presumably occurs because cellulose microfibril orientation is disrupted so that cellulose cannot constrain growth, and cell expansion becomes isotropic. We quantified the temporal correlations in cell growth fluctuations and found that oryzalin treatment did not affect the temporal correlations of growth in either wild type or *ftsh4-5* (Figure 7E). The mock treatment had little effect on wild type (Figures 7A, S7A, and S7B) or *ftsh4-5* sepal growth (Figures 7B, S7F, and S7G). Our results indicate that uncorrelated growth fluctuations are not restored in *ftsh4-5* mutants during the first few days following microtubule depolymerization.

To assess whether depolymerizing microtubules affects the spatial correlation of growth, we quantified the patchiness of the cumulative growth. Mock treatment does not affect the patchiness of cumulative growth (Figures 7F–7G and 7J). Oryzalin treatment increased patchiness in wild type and did not affect patchiness in *ftsh4-*5 (Figures 7H–7J). This indicates that depolymerizing microtubules cannot rescue the patchiness of growth in *ftsh4-5*. Instead, our results suggest that depolymerizing microtubules essentially further decreases microtubule dynamics, which increases patchiness in wild type, but is insufficient to further enhance patchiness in *ftsh4-5*. Thus, depolymerizing microtubules is not sufficient to decrease growth correlation and may instead enhance growth correlation.

## Discussion

There are spatial and temporal fluctuations in cell growth rates during Arabidopsis sepal development.^2^^,^^4^^,^^5^ Fluctuations in growth rate are not strongly correlated in space or time in the wild-type sepal, so the fluctuations average out over time and space. Consequently, the cumulative growth approaches the growth pattern developmentally specified by the overarching basipetal growth gradient, which ensures robustness of organ growth.^2^^,^^4^ By contrast, the *ftsh4-5* mutant has fluctuations in growth rate that are temporally correlated, which inhibits spatiotemporal averaging and causes growth to accumulate asymmetrically so that the organs have patches of both higher and lower cumulative growth.^2^^,^^4^ Consequently, although the basipetal growth gradient is present in *ftsh4*, the patches deviate from it, yielding variable sepal sizes and shapes. Various factors have been linked to correlations in growth rate, including microtubules,^8^ which indirectly affect the mechanical properties of the cell wall. Previously, it was found that decreasing ROS in *ftsh4-5* rescued the variability in mature organ size and shape.^2^ We find that elevated ROS promotes temporal correlations in growth fluctuations which inhibit spatiotemporal averaging of these growth fluctuations. Elevated ROS causes microtubules to become “crisscrossed,” and crisscrossed microtubules in *ftsh4-5* have increased stability. Cells with crisscrossed microtubules also exhibit temporally correlated growth fluctuations. However, depolymerizing microtubules was insufficient to decrease temporal correlations in growth fluctuations, and instead may increase spatial growth correlations, consistent with the hypothesis that microtubule dynamics are important for uncorrelated cell growth fluctuation.

### Complex relationship between growth direction and microtubule orientation

It is expected that cellulose microfibrils are assembled parallel to cortical microtubules, and that cell growth is perpendicular to cellulose microfibrils. Indeed, we found that the angle between cell growth direction and average microtubule direction is biased toward perpendicular. We also found that microtubule direction and growth direction are closer to perpendicular in cells that are growing faster than their neighbors. Further, the growth directions of cells growing slower than their neighbors are more influenced by the growth direction of the neighboring cells. This suggests that surrounding cells constrain growth and affect the relationship between microtubules and growth direction. However, the relationship is still not completely resolved as there are faster-growing cells with growth that is not perfectly perpendicular to the microtubule direction. There is the possibility that the timescale on which microtubules affect the growth direction is shorter than 24 h and we did not capture this effect, or that previously synthesized cell wall components have an influence on growth direction which is not accounted for here. The *csi1* mutant, in which cellulose synthase complexes are not tethered to the microtubules, demonstrates that previously synthesized cellulose has an effect on growth. The mutant has more anisotropic new cellulose, but similar anisotropy in total cellulose compared to wild type, and has similar growth anisotropy as wild type.^31^ We also imaged only the outer wall of each cell studied. The inner cell wall could contribute to the direction of cell growth. Further, the side (anticlinal) walls have been shown to be important for anisotropic growth and flattening of the organ,^30^ and they are also not accounted for here. Supracellular tensile stress also likely affects microtubule organization,^8^^,^^32^^,^^33^ and microtubules align better with the predicted direction of tensile stress than the cell growth direction in the boundary region of the shoot apical meristem.^32^ Altogether, our results suggest that although microtubules bias cell growth direction, the determinants of this direction are more complex and depend on both the cellular context within the tissue and the cell’s growth history.

### Relationship between microtubule dynamics and growth fluctuations

Here we found that depolymerizing microtubules was insufficient to reduce temporal correlations in growth rate fluctuations in the subsequent days. If cellulose microfibrils affect growth for a few days or longer, then the previous microtubule organization and cellulose microfibril organization would still affect the cells with no currently polymerized microtubules. Previously synthesized cellulose can also guide the cellulose synthase complexes in the absence of microtubules.^22^ This may explain why depolymerizing the microtubules did not affect temporal correlations in growth.

Depolymerizing microtubules caused an increase in spatial correlations in growth rate. Thus, it is also possible that microtubule dynamics are necessary for reducing correlations, and depolymerizing microtubules further decreases microtubule dynamics. There is evidence that microtubule dynamics can reduce growth rate correlations; increased growth of a differentiating trichome cell causes increased tension, increased microtubule anisotropy, and decreased cell growth rate in the surrounding cells.^17^ Microtubule dynamics promote anisotropic arrangements^34^ and the ability to change the orientation of microtubules over time.^8^ Perhaps changes in microtubule anisotropy and direction create changes in cell wall properties over time and space, leading to uncorrelated fluctuations in cell growth rates. On the other hand, more stable microtubules may not generate changes in cell wall properties over time and space, leading to more correlated fluctuations of cell growth rates.

### Microtubule organization could be a consequence of tensile stress in the cell

Cells in *ftsh4-5* sometimes become lobed (indented in places to create a more complex shape).^35^ Usually, these cells are in the slow-growing patches that have increased temporal correlation of growth fluctuations. Leaf epidermal cells are also lobed to the extent that they are described as “jigsaw puzzle piece-shaped.” The development of lobes mediates tensile stress by transferring it from the center of the cell to the indented regions of the cell.^35^^,^^36^ The indented regions have increased microtubule anisotropy, which is likely because microtubules align parallel to cell shape-derived stress.^36^ Therefore, it is possible that microtubules in sepal cells are influenced by stress created by cell geometry, especially in lobed *ftsh4-5* cells.

### ROS in sepal development

The elevated ROS levels in *ftsh4-5* could be disrupting a normal role of ROS in development. Although ROS is toxic at high levels, normal levels of ROS are important for redox biology and many cellular functions, and thus ROS levels are tightly regulated.^16^ ROS localization has a spatial pattern in Arabidopsis sepals, in which ROS first appears at the tip of the sepals and then progresses toward the base.^2^ This suggests that ROS also has a normal, regulated role in sepal maturation. ROS signaling is not the only mechanism by which metabolism and growth are linked. Nutrient levels and sensing also affect growth.^37^ The TOR complex is involved in sensing energy, and in Arabidopsis, decreased TOR activity affects protein translation and results in variable localization of auxin and cytokinin, which disrupts the timing and localization of sepal initiation.^38^ Thus, general cellular functions that occur throughout the life cycle can also have a role in growth heterogeneity and developmental robustness.

### Limitations of the study

The increased stability and thicker appearance of crisscrossed microtubules in *ftsh4-5* suggest that they are bundled. In sepals, the curvature of the tissue limits the quality of microtubule imaging due to the curvature of microtubules in the z plane, and because the 3D structure of a flower bud limits the options for mounting samples for imaging. Therefore, we were unable to observe microtubule dynamics at a resolution that would have allowed us to observe bundling of individual microtubules directly. However, aspects of *ftsh4-5* microtubule dynamics are similar to those observed for bundling, such as a thicker appearance and increased stability.^39^ Crisscrossed microtubules in *ftsh4-5* cross over each other at large angles so that many cross over each other to form a star shape. Typically, in wild type, microtubules that cross over at large angles lead to severing, whereas crossing over at shallow angles leads to bundling, and this leads to organization.^34^ MAP65-1 mediates microtubule bundling, and this type of bundling is protective against severing by KATANIN1.^40^ Changing the rate of severing or the stability of microtubules also changes microtubule organization.^34^^,^^41^ Therefore, it is possible that MAP65-1 activity, or the activity of a similar protein, could create the crisscrossed pattern in *ftsh4-5* by inhibiting the severing of microtubules. There is also evidence, in maize, that ROS increases the expression of MAP65-1.^42^ Thus, we hypothesize that increased stability of *ftsh4-5* microtubules is likely due to increased bundling, and that bundling may lead to the disorganized crisscrossed microtubule pattern. In the future, it will be interesting to test whether MAP65-1 or other MAPs contribute to crisscrossed microtubules in *ftsh4-5*.

The two molecular markers we have used to visualize the microtubules reveal slightly different microtubule patterns in the *ftsh4-5* mutant, making it unclear which pattern more accurately represents the mutant phenotype. *ftsh4-5 GFP-MBD* has cells with longitudinal microtubules that become crisscrossed, whereas *ftsh4-5 GFP-TUB6* has microtubules that are mostly longitudinal and do not become crisscrossed during the developmental stages imaged. Note that both of these patterns are different from the transverse or isotropic microtubules observed in wild type. It was previously reported that *GFP-MBD* does enhance bundling/crisscrossed microtubules after compression, but that both the *GFP-MBD* and *GFP-TUB6* lines recover similarly and return to the normal microtubule organization.^24^ It is possible that *GFP-MBD* might artificially enhance the short-term differences in stability in the propyzamide experiment. The longitudinal microtubules in *ftsh4-5* cells at earlier stages were not noticeably more stable compared to microtubules in wild-type cells. If the enhanced stability of the microtubules is artificial, then perhaps the orientation of microtubules is affecting cell growth rate or cell growth fluctuations rather than the dynamics.

Another limitation of the study is the low sample size of the live imaging experiments, with only three replicates per genotype, because live imaging and lineage tracking are labor-intensive. Given the variable phenotype of *ftsh4-5*, this adds some uncertainty to the degree of rescue by *CAT2oe*.

Despite these limitations, our findings highlight a relationship between ROS, microtubules, and cell growth heterogeneity that underlies robust morphogenesis.

## Resource availability

### Lead contact

Requests for further information and resources should be directed to and will be fulfilled by the lead contact, Adrienne H.K. Roeder (ahr75@cornell.edu).

### Materials availability

Seeds for key plant lines are available from the Arabidopsis Biological Resource Center (ABRC). Accessions are listed in the key resources table. The plasmid for *CAT2oe* and any other seeds for plant lines are available upon request to the lead contact without restriction.

### Data and code availability

• All data and meshes from live imaging series, microtubule images, and ilastik files have been deposited in Zenodo and are publicly available at https://doi.org/10.5281/zenodo.18944692
• All original code has been deposited in Zenodo and is publicly available at https://doi.org/10.5281/zenodo.18944692
• Any additional information required to reanalyze the data reported in this paper is available from the lead contact upon request.

## Acknowledgments

We thank Byron Rusnak, Michelle Heeney, Lilijana Sarabia Oliver, and Si Chen for helpful comments on the manuscript. We thank Arezki Boudaoud for conversations and feedback. We thank Ram Dixit for discussing concepts and protocols for depolymerizing microtubules. Research reported in this publication was supported by the 10.13039/100000057National Institute of General Medical Sciences of the National Institutes of Health under award numbers R01GM134037 and R35GM158190 (to A.H.K.R.), the 10.13039/100000936Gordon and Betty Moore Foundation post-doctoral fellowship award #2919 (to F.B.), and funding from the 10.13039/501100004189Max Planck Society (to F.B.). The content is solely the responsibility of the authors and does not represent the views of the National Institutes of Health and other funders.

## Author contributions

Conceptualization: I.B., L.H., and A.H.K.R.; data curation: I.B.; formal analysis: I.B. and F.B.; funding acquisition: F.B. and A.H.K.R.; investigation: I.B., E.S., and L.H.; methodology: I.B. and A.S.; project administration: A.H.K.R.; supervision: A.H.K.R.; visualization: I.B.; writing – original draft: I.B.; writing-review and editing: I.B., F.B., A.S., E.S., L.H., and A.H.K.R.

## Declaration of interests

The authors declare no competing interests.

## STAR★Methods

### Key resources table

**Table:** 

REAGENT or RESOURCE	SOURCE	IDENTIFIER
**Chemicals, peptides, and recombinant proteins**		

3,3′-diaminobenzidine (DAB)	Krackeler (Sigma)	D8001-1G
Nitroblue tetrazolium (NBT)	Krackeler (Sigma)	45-93862-100 MG
Propyzamide	Thermo	P23741G
Oryzalin	Krackeler (Sigma)	45-36182-100 MG-EA
3-Amino-1,2,4-triazole (3-AT)	Sigma Aldrich	A8056
LR clonase II	Invitrogen	11791020
DNase I (RNase-free)	NEB	M0303L
Invitrogen Superscript II Reverse Transcriptase	Thermo Fisher	18064014

**Critical Commercial Assays**		

RNeasy Plant Mini Kit	Qiagen	74904

**Deposited data and code**		

Arabidopsis sepal live-imaging datasets CAT2 and oryzalin, 3-AT microtubule images, microtubule screenshots, propyzamide images, tubulin 6 marker images, and microtubule crisscross analysis images and code.	Zenodo	https://doi.org/10.5281/zenodo.18944692

**Experimental models: Organisms/strains**		

*pUBQ10*:*:GFP-MBD*	Lab of Oliver Hamant	
*ftsh4-5 pUBQ10*:*:GFP-MBD*	Our lab,	N/A
*pUBQ10*:*:GFP-MBD pUBQ10*:*:mCherry-RCI2A*	Our lab, also available from ABRC	CS743126
*pUBQ10*:*:GFP-MBD pUBQ10*:*:mCherry-RCI2A p35S*:*:CAT2*	Our lab, also available from ABRC	CS743128, CS743129, CS743130
*ftsh4-5 pUBQ10*:*:GFP-MBD pUBQ10*:*:mCherry-RCI2A*	Our lab, also available from ABRC	CS743127
*ftsh4-5 pUBQ10*:*:GFP-MBD pUBQ10*:*:mCherry-RCI2A p35S*:*:CAT2*	Our lab, also available from ABRC	CS743131, CS743132, CS74133
*p35S*:*:RFP-TUB6 pUBQ10*:*:GFP-MBD*	Our lab, also available from ABRC	CS743134
*ftsh4-5 p35S*:*:RFP-TUB6 pUBQ10*:*:GFP-MBD*	Our lab, also available from ABRC	CS743135

**Recombinant DNA**		

pUBQ10:GFP-MBD	Lab of Oliver Hamant	N/A
pUBQ10:mCherry-RCI2A	Our lab	pMZ12
p35S::CAT2	Our lab	pBB7
p35S::RFP-TUB6	Lab of Ram Dixit	N/A

**Software and algorithms**		

MorphoGraphX	^43^^,^^44^	N/A
ilastik	^25^	N/A

**Oligonucleotides**		

CAT2 qPCR Pair 1 Forward: 5′ GCACAGGGACGAGGAGGTTA 3′	Our lab	oBB57
CAT2 qPCR Pair 1 Reverse: 5′ GCAGGCGGAGTTGGATACTT 3′	Our lab	oBB58
CAT2 qPCR Pair 2 Forward: 5′ TGGAAAACGTGAGAGGTGCAT 3′	Our lab	oBB59
CAT2 qPCR Pair 2 Reverse: 5′ TGCGGATTTCATGCGTGATG 3′	Our lab	oBB60
CAT2 qPCR Pair 3 Forward: 5′ TCTTCAACCTGTTGGACGTATG 3′	Our lab	oLH275
CAT2 qPCR Pair 3 Reverse: 5′ ATAGGAGAAGACACGGGTTTGA 3′	Our lab	oLH276
Genotyping primer in p35S: 5′ CAACCACGTCTTCAAAGC 3′	Our lab	oBB27
Genotyping primer in CAT2 CDS: 5′ GATAACGGTGGAGAACCG 3′	Our lab	oBB28
dCAPs forward genotyping primer for *ftsh4-*5 (Nco1 cuts WT): 5′ AGAAAGGACTCACTTTAAAGAACAGCCATG 3′	Our lab,^2^	oLH168
dCAPs reverse genotyping primer for *ftsh4-*5 (Nco1 cuts WT): 5′ TCCTCTGTCCTCGATAAGAGCTCC 3′	Our lab,^2^	oLH169

### Experimental model and study participant details

*Arabidopsis thaliana* plants were grown in a Percival model AR-1115L3 growth chamber at 22°C, in 60% humidity with a 16 hour light/8 hour dark cycle. Plants were illuminated with Philips F32T8/TL741 700 series 32-watt fluorescent light bulbs at 100 μmol m^2^ s^-1^ intensity. The soil was Lambert LM-111 All Purpose Mix. Seeds were placed on moist soil, stratified at 4 degrees for 2-14 days before they were moved into the growth chamber for germination. The plants were bottom watered using a nutrient solution made from Jack’s 21-5-20 fertilizer with Epsom salt, mixed in purified reverse osmosis water at a 1:100 dilution, delivering 150 PPM of nitrogen. The stock solution was prepared by dissolving 4.02 lbs. fertilizer and 3.75lbs Epsom salt in a 10-gallon tank.

### Method details

#### Plant material

The *Arabidopsis thaliana* accession Col-0 plants are used as wild type and all mutants are in Col-0 background as well. Isolation of the *ftsh4-5* mutant is described in Hong et al. 2016. The membrane marker is the transgene *pUBQ10::mCherry-RCI2A*. The microtubule marker is the transgene *pUBQ10::GFP-MBD* where MBD is the microtubule binding domain of MAP4, and the transgene came from the lab of Olivier Hamant. The transgenes were crossed into *ftsh4-5*. The original *p35S::CATALASE2* plants from Hong et al. 2016 were silencing expression of the transgene when crossed into the plants with microtubule and membrane markers. Thus, we did an LR reaction (LR clonaseII) with *CAT2* inserted into pDONR201 with pK7WG2 to make a new Kanamycin resistant *p35S::CAT2* (also named pBB7). We transformed *p35S::CAT2* into wild-type and *ftsh4-5* plants with both the membrane and microtubule markers. Individual T1s were used for experiments.

#### Genotyping, plant selection and qPCR

Genotyping of *ftsh4-5* was done as described in Burda et al. 2024 and Hong et al. 2016. Membrane and microtubules marker plants were selected by screening for fluorescence. *p35S::CATALASE2* was selected by germination on media with kanamycin. The media contained of 2.2g/L Murashige and Skoog, 0.5g/L MES, 5g/L sucrose, the pH was brought to 5.7 with KOH, then 10g/L phytoagar was added, and after autoclaving kanamycin was added to the concentration of 50μg/ml. Leaf tissue was collected from individual T1 plants used for imaging and ROS stains, and then mRNA was extracted using the RNeasy Plant Mini Kit using the RLT buffer and beta-mercaptoethanol. Then cDNA was synthesized from the mRNA by doing a DNase treatment (NEB DNase1(RNase-free) catalog number M0303L) and then forward strand synthesis (Invitrogen Superscript II Reverse Transcriptase catalog number 18064014), and then used for qPCR. Three separate primer pairs targeting *CATALASE2* were used. Primer pair one is: 5′ GCACAGGGACGAGGAGGTTA 3′ and 5′ GCAGGCGGAGTTGGATACTT 3’. Primer pair two is: 5′ TGGAAAACGTGAGAGGTGCAT 3′ and 5′ TGCGGATTTCATGCGTGATG 3’. Primer pair three is: 5′ TCTTCAACCTGTTGGACGTATG 3′ and 5′ ATAGGAGAAGACACGGGTTTGA 3’. Fold change of each replicate was normalized to the average expression level of the three wild type replicates. Genotyping of the T2s to confirm segregation of *p35S::CATALASE2* was done with the forward primer in the 35S promoter (5′ caaccacgtcttcaaagc 3′) and the reverse primer in the *CAT2* coding sequence (5′ GATAACGGTGGAGAACCG 3′).

#### Images of phenotypes and ROS stains

Images of flower morphology and ROS stains were taken with an Excelis 4K camera mounted on a Zeiss Stemi 508 stereomicroscope. Inflorescences stained for ROS were submerged in the same solution used for bleaching chlorophyll from the samples (3:1:1 ratio of ethanol: acetic acid: glycerol), arranged to spread out the flowers, and had a coverslip placed on top.

#### ROS stains

3,3′-diaminobenzidine (DAB) and nitroblue tetrazolium (NBT) were used to stain for hydrogen peroxide and superoxide, respectively. Staining solutions and the bleaching solution were made as described in Hong et al. 2016. Inflorescences were stained with DAB for 5 hours and with NBT for 24 hours, including vacuum infiltration for about 25 min.

#### Microscopy and image analysis

Inflorescences were dissected and mounted in apex culture media.^45^ Media containing 2.3 g/L Murashige and Skoog, 1% sucrose and 0.1% MES was brought to a pH of 5.8 with KOH, and agarose was added to a concentration of 1.2%. After autoclaving, media was supplemented with vitamins (final concentration of 100 μg/ml myoinositol, 1 ng/ml nicotinic acid, 1 ng/ml pyridoxine hydrochloride, 1 ng/ml thiamine hydrochloride, 2 ng/ml glycine). Plants then grew in 16 h light/8 h dark conditions on the media. Plants were switched to new media every 2-3 days. Abaxial sepals were imaged because they face outwards, making them the most accessible for imaging.

For the 24 hr live time lapse imaging, plants with the transgenes *pUBQ10::GFP-MBD* and *pUBQ10::mCherry-RCI2A* were dissected two days before imaging. Images were taken with a Leica Stellaris 5 using a 25X water dipping objective with an NA of 0.95 (HC FLUOTAR L VISIR 25X/0.95 WATER). A 488 laser with a power of 0.7 and a gain of 75, detecting wavelength of 494-550, was used to image GFP-MBD. A 561 laser with a power of 0.7 and a gain of 100, detecting wavelength of 582-607, was used to image mCherry-RCI2A. Signal from anthocyanin was collected at wavelengths 647-656 with a gain of 100 in a third channel. Channels were imaged in the same track, with line averaging of 2 and a scanning speed of 400, and bidirectional scanning. Images were a format of 1024x1024 pixels and 8 bit. The zoom was adjusted to fit the sepal and ranged from 2.25-3 on the first day of imaging and 1.6-2 on the last day of imaging. The z-step was 0.2μm. Inflorescences were positioned at an angle before each image and imaged once every 24 hrs.

MorphoGraphX was used to analyze cell growth and cortical microtubules. To remove red fluorescence from anthocyanin which interfered with the mCherry-RCI2A signal, the anthocyanin signal was added to itself to be twice as bright, and then subtracted from the mCherry-RCI2A channel. Then a neural network (VijayanUNET) was run on the ‘cleaned’ mCherry-RCI2A channel to predict locations of the cell wall, which creates a stack with a brighter and more continuous surface. This new stack was then used with an edge detect signal threshold to detect the surface. This was used to create the mesh, and then the signal from the cleaned mCherry-RCI2A was projected onto the mesh and used for segmentation. Cell lineages were tracked across time points, and then cell area growth and principal directions of cell growth, and anisotropy of growth direction were calculated. The cell neighborhoods were also saved. The proximal-distal axis was created by selecting cells at the tip of the sepal, creating a distance heat map from the selected cells (and saving the heat map), and then generating a custom axis from the heat map directions, and smoothing the axis. The principal directions of cell growth were added to the attribute map, and then the angle between the custom axis and the principal directions of growth was calculated. Then the microtubule signal was projected onto the mesh, cells with blurry signal were deleted, and this new version of the mesh was saved separately. The fibril directions were saved as a cell axis and added to the attribute map. Then the principal directions of growth were loaded onto the new mesh and the angle between growth directions and fibril directions was calculated. Then the distance heat map was loaded onto the new mesh, a proximal distal axis was created like before, and the angle between the fibril directions and the custom axis was calculated (which is the angle of the microtubules relative to the proximal-distal axis). Then to better visualize the microtubule signal at the surface of the image, the channel with the microtubule signal was loaded, and then the mesh was used to “annihilate” the signal everywhere except 1μm above and 1μm below the mesh. This annihilated image was used for screenshots of microtubule signal.

For the CATALASE inhibitor treatment (treatment details are described below), images were taken with a Leica Stellaris 5 using a 25X water dipping objective with an NA of 0.95 (HC FLUOTAR L VISIR 25X/0.95 WATER). A 488 laser with a power of 1 and a gain of 75, detecting wavelength of 494-550, was used to image GFP-MBD. A 561 laser with a power of 0.2 and a gain of 100, detecting wavelength of 582-607, was used to image propidium iodide. Channels were imaged in the same track, with line averaging of 2 and a scanning speed of 400, and bidirectional scanning. Images were a format of 1024x1024 pixels and 8 bit. Voxel size was x=0.0865799μm, y=0.0865799μm, z=0.2μm. MorphoGraphX was used to process the images. A neural network was run on the propidium iodide channel to predict locations of the cell wall, which creates a stack with a brighter and more continuous surface. This new stack was then used with an edge detect signal threshold to detect the surface. This was used to create the mesh, and then the signal from the propidium iodide was projected onto the mesh and used for segmentation. Then to better visualize the microtubule signal at the surface of the image, the channel with the microtubule signal was loaded, and then the mesh was used to “annihilate” the signal everywhere except 1μm above and 1μm below the mesh and used for screenshots of microtubule signal.

For the propyzamide treatments, plants with the *pUBQ10::GFP-MBD* transgene were dissected two days before imaging and placed on media. Images were taken with a Zeiss LSM 710 using a 20× water dipping objective with an NA of 1.0 (W Plan-APOCHROMAT20×.1.0DIC(US)VIS-IR). A 488 laser with a power of 25 and a gain of 750, detecting wavelength of 493-586, was used to image GFP-MBD. A 594 laser with a power of 2 and a gain of 670, detecting wavelengths of 599-641, was used to image propidium iodide. Channels were imaged in the same track, with line averaging of 4. Images were a format of 512x512 pixels and 16 bit. Voxel size was x=0.1383776, y=0.1383776, z=0.2 μm. For image processing, signal from propidium iodide is used with a neural network to predict the location of cell walls, and then the prediction is used with an edge detect signal threshold to detect the surface. This is used to create the mesh, and then the signal from the neural network predict is projected onto the mesh and used for segmentation. To better visualize the microtubule signal at the surface of the image, the channel with the microtubule signal was loaded, and then the mesh was used to “annihilate” the signal everywhere except 1μm above and 1μm below the mesh. Then the before and after treatment time points were lineage tracked, and cells are scored based on the microtubule signal.

For the 5 min and 10 sec time lapse imaging of microtubules, plants with the transgenes *pUBQ10::GFP-MBD* and *pUBQ10::mCherry-RCI2A* were dissected two days before imaging. Images were taken with a Leica Stellaris 5 using a 25X water dipping objective with an NA of 0.95 (HC FLUOTAR L VISIR 25X/0.95 WATER). A resonant scanner with a speed of 8000 was used with bidirectional scanning and line averaging of 16. A 488 laser with a power of 3 and a gain of 150, detecting wavelengths of 494-550, was used to image GFP-MBD. Images were a format of 1024x1024 pixels and 8 bit. Voxel size was x=0.0270563μm, y=0.0270563μm, z=0.2 μm for the stage 5 (earlier stage) sepals, and x=0.432899μm, y=0.432899μm, z=0.2 μm for the stage 7 (later stage) sepals. For the 10 sec images, voxel size was x= 0.0240502μm, y= 0.0240502μm, z=0.3 μm. For the 5 min time lapse, images were set up and taken every 5 min for 65 min, and junctions between cells were used as landmarks to align images as best as possible. For the 10 sec times series, 20 stacks of 9 slices were taken which took roughly 10 seconds each. In ImageJ, max projections were combined into a stack, and then the plugin HyperStack (rigid body) was used to register the images. To make the merges for the 5 min images, after registration, the time points were split into images, and the 0 min and 5 min time points were merged.

For FRAP, plants with the transgenes *p35S::RFP-TUB6* and *pUBQ10::mCherry-RCI2A* were dissected two days before imaging. Images were taken with Leica Stellaris 5 FRAP mode using a 25X water dipping objective with an NA of 0.95 (HC FLUOTAR L VISIR 25X/0.95 WATER). A resonant scanner with a speed of 8000 was used with bidirectional scanning and line averaging of 16. A 488 laser with a power of 3 and a gain of 150, detecting wavelengths of 494-550, was used to image GFP-MBD. Images were a format of 1024x1024 pixels and 8 bit. Voxel size x= 0.0240502μm, y= 0.0240502μm, z=0.3 μm. After bleaching, 20 stacks were taken, which took roughly 10 sec per stack, and each stack was 9 slices. For quantification, MAX intensity projections were created and then ROI’s were drawn both at the bleached section and a section with unbleached signal, and the average intensity in the ROI was exported for each frame. Then the difference between the bleached ROI and unbleached ROI was found for each frame. Then the minimum difference and maximum difference was found for each replicate. Then for each frame, signal was normalized by the difference at a given frame minus the maximum difference divided by the minimum difference minus the maximum difference. Then these values were averaged for all frames of a given time point for each genotype. Error bars represent standard error.

#### CATALASE inhibitor treatment

Inflorescences with the microtubule marker *pUBQ10::GFP-MBD* were dipped in 200 μM 3-AT in a HEPES buffer with 0.02% Silwet and pH of 7 or a mock treatment of just HEPES buffer with 0.02% Silwet and pH of 7 once per day for 4 days. On day 5, inflorescences were either imaged or stained for hydrogen peroxide. For the inflorescences that were imaged, they were dissected, placed on apex culture media (described above) in a tilted position with flowers of about stage 6 at the top stained with 1.87 mM propidium iodide for 4 min by filling the petri dish with the solution with the inflorescences mounted in media, then the stain was removed, and inflorescences were rinsed twice by placing water on top of the inflorescences in the media. Images were taken 5-10 min after the last rinse, which is enough time for the position of the sample to stop moving. This process was performed one at a time for each inflorescence to minimize effects of ROS created by dissection. For the inflorescences stained for hydrogen peroxide, the DAB stain solution was made as described above, and inflorescences were stained for 25 hrs.

#### Propyzamide treatment

For the propyzamide treatment, propyzamide was added to the apex culture media (described above) at the same time as the vitamins are added to the media. A concentrated stock of 10mM of propyzamide in DMSO was made, and then added to the media to create a 200μM final concentration. Propyzamide concentrations of 20μM, 100μM, and 200μM were tested. Media with 200μM propyzamide often caused complete depolymerization of microtubules after 24 hours. Sepals on 200μM propyzamide media were imaged once every 30 min, starting at 15 min after being moved to the media. A noticeable amount of depolymerization occurred between 15 min and 45 min. Between 45 min and 75 min the microtubules became short and thick, which could indicate some rate of polymerization. From 75 min to 225 min the rate of depolymerization was slower, which may again indicate some rate of polymerization. Therefore, a 30min treatment of 200μM propyzamide was chosen as optimal for causing depolymerization with minimal effects of re-polymerization.

Plants were dissected two days before the experiment to allow for recovery from dissection. One at a time, inflorescences were stained in 1.87 mM propidium iodide with 0.02% Silwet for 4 min in a lid of an Eppendorf tube, then rinsed by being placed in the lid of an Eppendorf tube with water, placed in normal apex culture media, and placed in a tilted position so that a stage 5-6 sepal was most visible. Then water was added to the petri dish for about 10 min before imaging to allow the inflorescence to settle into the media and water. Sepals were imaged, moved to propyzamide media in the same tilted position, water was added to the petri dish again, and imaged for a second time after 30 min on the propyzamide media.

#### Oryzalin treatment

For the oryzalin treatment, oryzalin was added to the apex culture media (described above) at the same time as the vitamins are added to the media. A concentrated stock of 84.2mM of oryzalin in DMSO was made, and then added to the media to create a 100 μM final concentration. This concentration of oryzalin was enough to begin depolymerizing microtubules within 24 hours, and completely depolymerize microtubules within 48 hours. Higher concentrations precipitated in the media. Plants were dissected and then placed on normal media for one day to recover from dissection. After recovering from dissection for one day, inflorescences were moved to media with oryzalin or a DMSO mock treatment (and imaged on the third day, about 48 hours after dissection). Plants were moved to new media halfway through the imaging series.

### Quantification and statistical analysis

#### Analysis of temporal and spatial heterogeneity (patchiness)

Analysis of heterogeneity was done as described in Burda et al. 2024.

#### Quantification of microtubule organization on a subcellular scale

Pixel classification in ilastik^25^ was used to classify microtubules as organized or crisscrossed. The images used were screenshots of ‘annihilated’ microtubule signal (described above), and the background and any blurred signal at the periphery was cropped out of the images, and then images were normalized for signal intensity. For Figure 3, a small subset of pixels on a few images in the data set were used for training, and then the rest of the pixels were batch processed. Although not completely unbiased, it is a way to classify visual impressions blindly and reproducibly. For Figure 4, the images were batch processed using the same classification system from Figure 3.

#### Statistical analysis

Pairwise *p* values for the temporal heterogeneity and patchiness scores in Figures 1 and 7 were obtained using z-test for correlation coefficients. Figure 3 *p* values were obtained using a T-test between genotype pairs using data from all replicates and days. Figure 4 *p* values were obtained using student t-tests. Figure 6 *p* values were obtained using student t-tests. All of the statistical details of experiments can be found in the figure legends.
