# Supplementary material for: ROS inhibits microtubule dynamics and cell growth heterogeneity during Arabidopsis sepal morphogenesis
Source: iScience. 2026 Jun 23;29(7):116426. doi: 10.1016/j.isci.2026.116426 (PMC13319952; doi:10.1016/j.isci.2026.116426)
Supplement: Document S1. Figures S1–S8 [file mmc1.pdf]

**iScience, Volume *29***

## Supplemental information

# ROS inhibits microtubule dynamics

# and cell growth heterogeneity

# during Arabidopsis sepal morphogenesis

**Isabella Burda, Fridtjof Brauns, Aaron Shipman, Emily Shapland, Lilan Hong, and Adrienne H.K. Roeder**

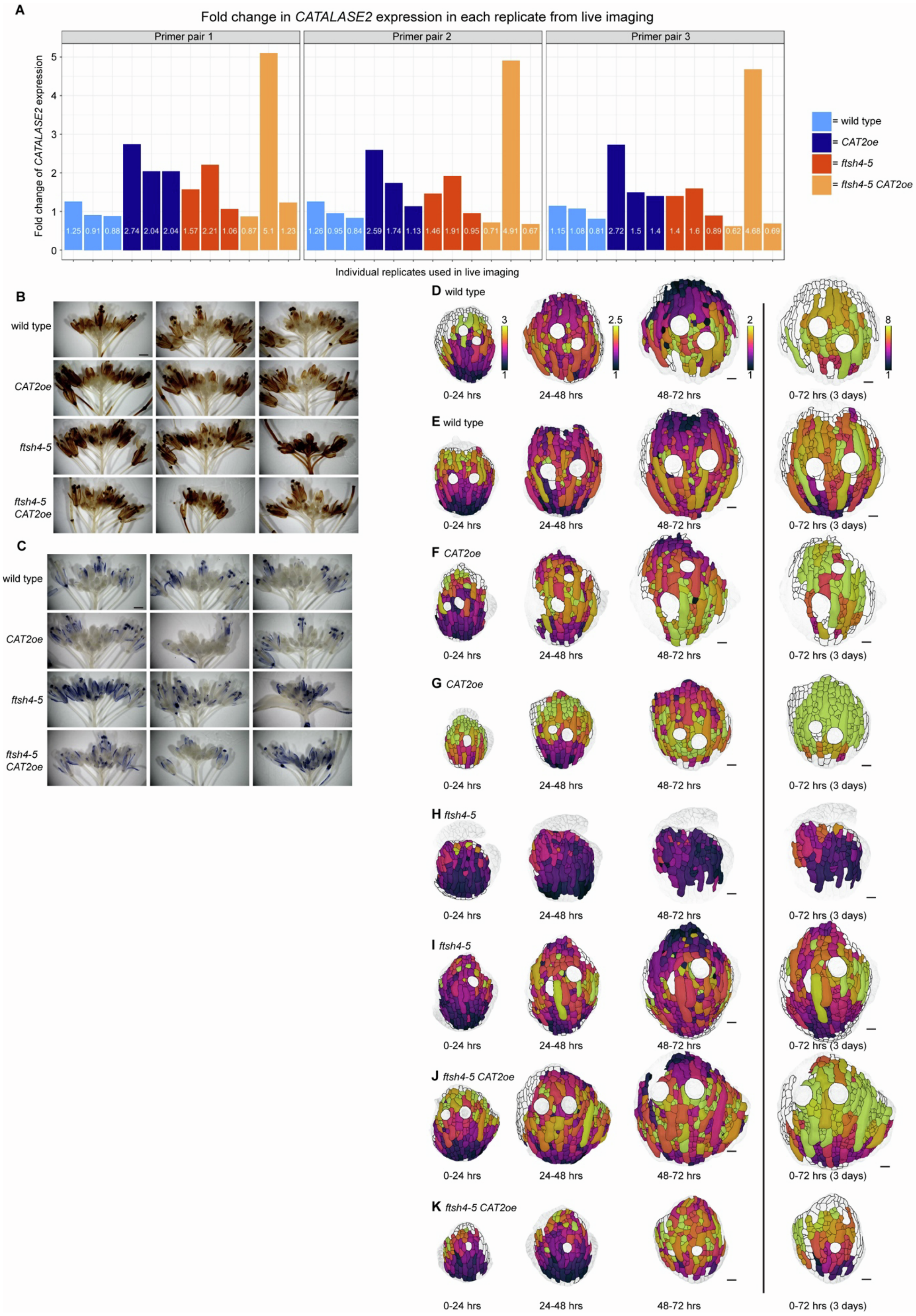
A
Fold change in CATALASE2 expression in each replicate from live imaging
Primer pair 1
Primer pair 2
Primer pair 3
Fold change of CATALASE2 expression
0
1
2
3
4
5
1.25 0.91 0.88 2.74 2.04 2.04 1.57 2.21 1.06 0.87 5.1 1.23
1.26 0.95 0.84 2.59 1.74 1.13 1.46 1.91 0.95 0.71 4.91 0.67
1.15 1.08 0.81 2.72 1.5 1.4 1.4 1.6 0.89 0.62 4.68 0.69
Individual replicates used in live imaging
= wild type
= CAT2oe
= ftsh4-5
= ftsh4-5 CAT2oe
B
wild type
CAT2oe
ftsh4-5
ftsh4-5 CAT2oe
C
wild type
CAT2oe
ftsh4-5
ftsh4-5 CAT2oe
D wild type
3
1
2.5
1
2
1
8
1
E wild type
F CAT2oe
G CAT2oe
H ftsh4-5
I ftsh4-5
J ftsh4-5 CAT2oe
K ftsh4-5 CAT2oe
0-24 hrs
24-48 hrs
48-72 hrs
0-72 hrs (3 days)

Figure S1: **ROS inhibits growth heterogeneity, Related to Figure 1.** (A) Fold change in *CATALASE2* expression from QPCR of tissue from the same plants used for individual live imaging replicates. (B) Inflorescences stained for hydrogen peroxide. Scale bars are 0.5 mm. (C) Inflorescences stained for superoxide. Scale bars are 0.5 mm. I-L: Remaining replicates of live imaging. Cell area growth represented as a ratio of area at the later time point divided by area at the earlier time point, projected on the later time points over 24-hour intervals and cumulative over 3 days for (D-E) wild type, (F-G) *CAT2oe,* (H-I) *ftsh4-5*, and (J-K) *ftsh4-5 CAT2*. Scale bars are 20µm.

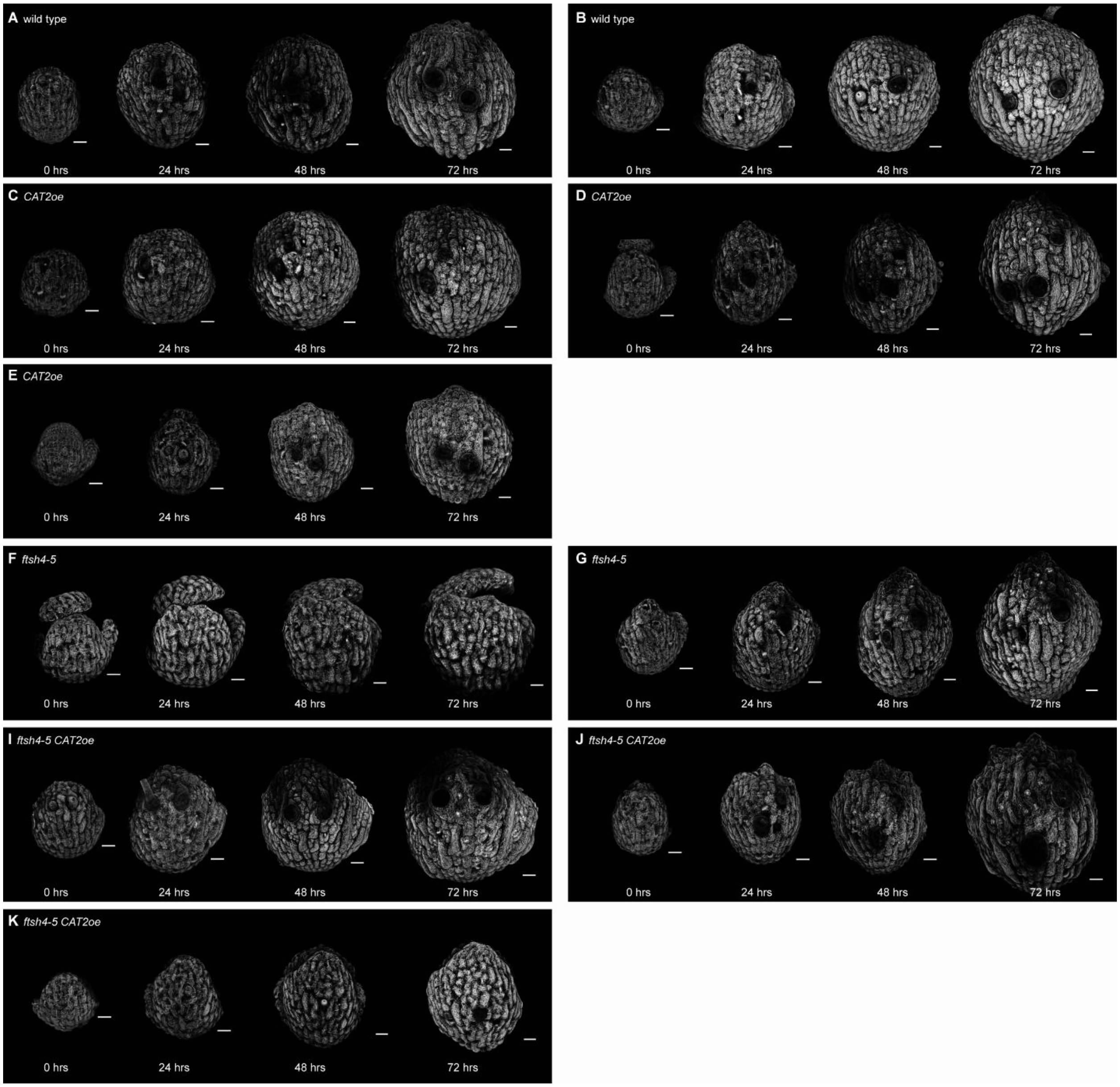


Figure S2: **Microtubule arrangement is different in *ftsh4-5* and rescued in *ftsh4-5 CAT2oe,* Related to Figure 2.** A-J: Microtubule signal from the live time lapse imaging of sepal development. Other two replicates of wild type (A-B), all three replicates of *CAT2oe* (C-E), other two replicates of *ftsh4-5* (F-G), and all three replicates of *ftsh4-5 CAT2oe* (I-K). Scale bar is 20μm.

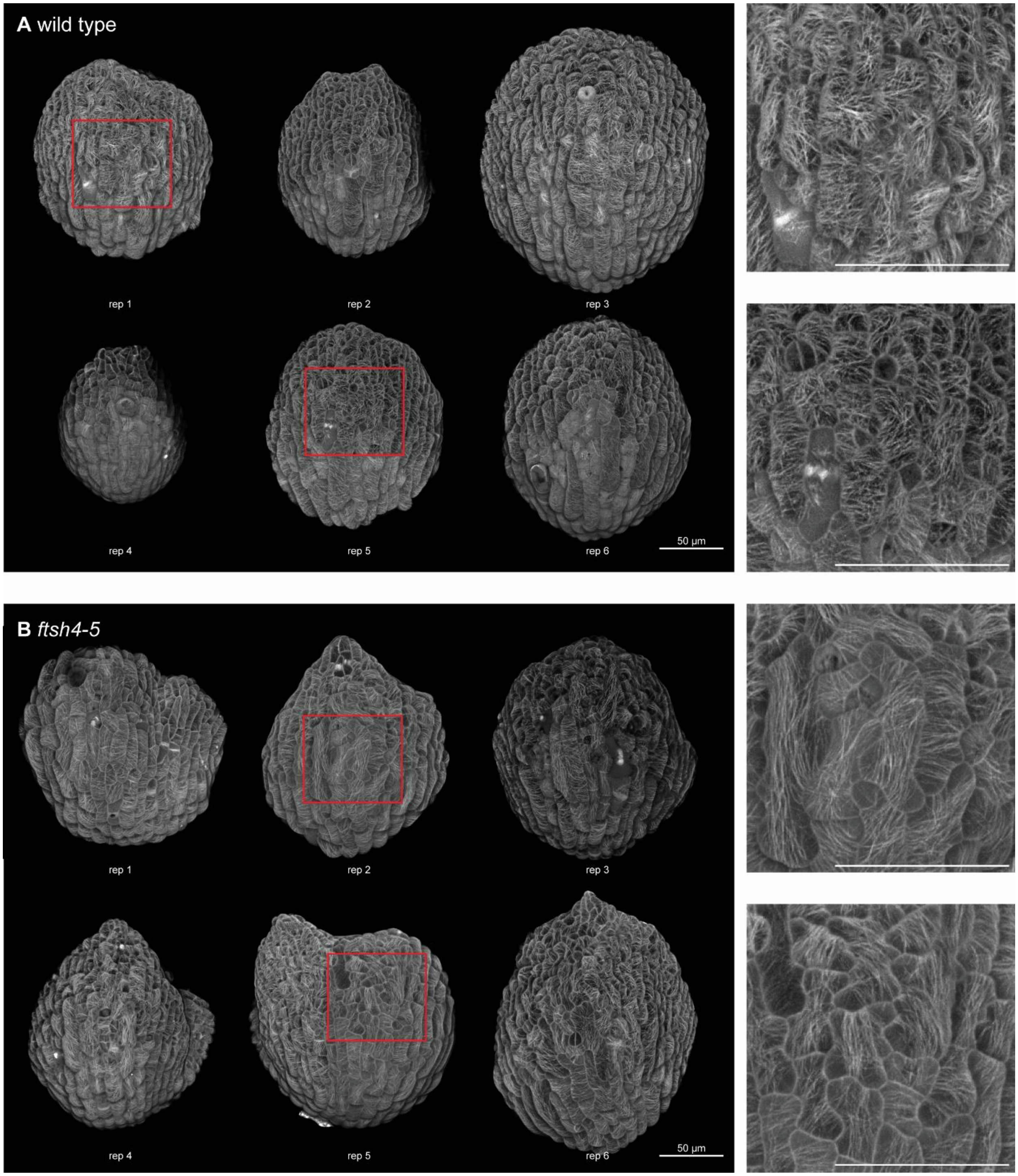


Figure S3: ***ftsh4-5* has longitudinal microtubules with the TUB6 marker, but the same variable organ sepal morphology, Related to Figure 2.** A-B: *p35S::RFP-TUB6* and *pUBQ10::mCherry-RCI2A* signal in wild type (A) and *ftsh4-5* (B). Red boxes indicate location of magnified images on the right.

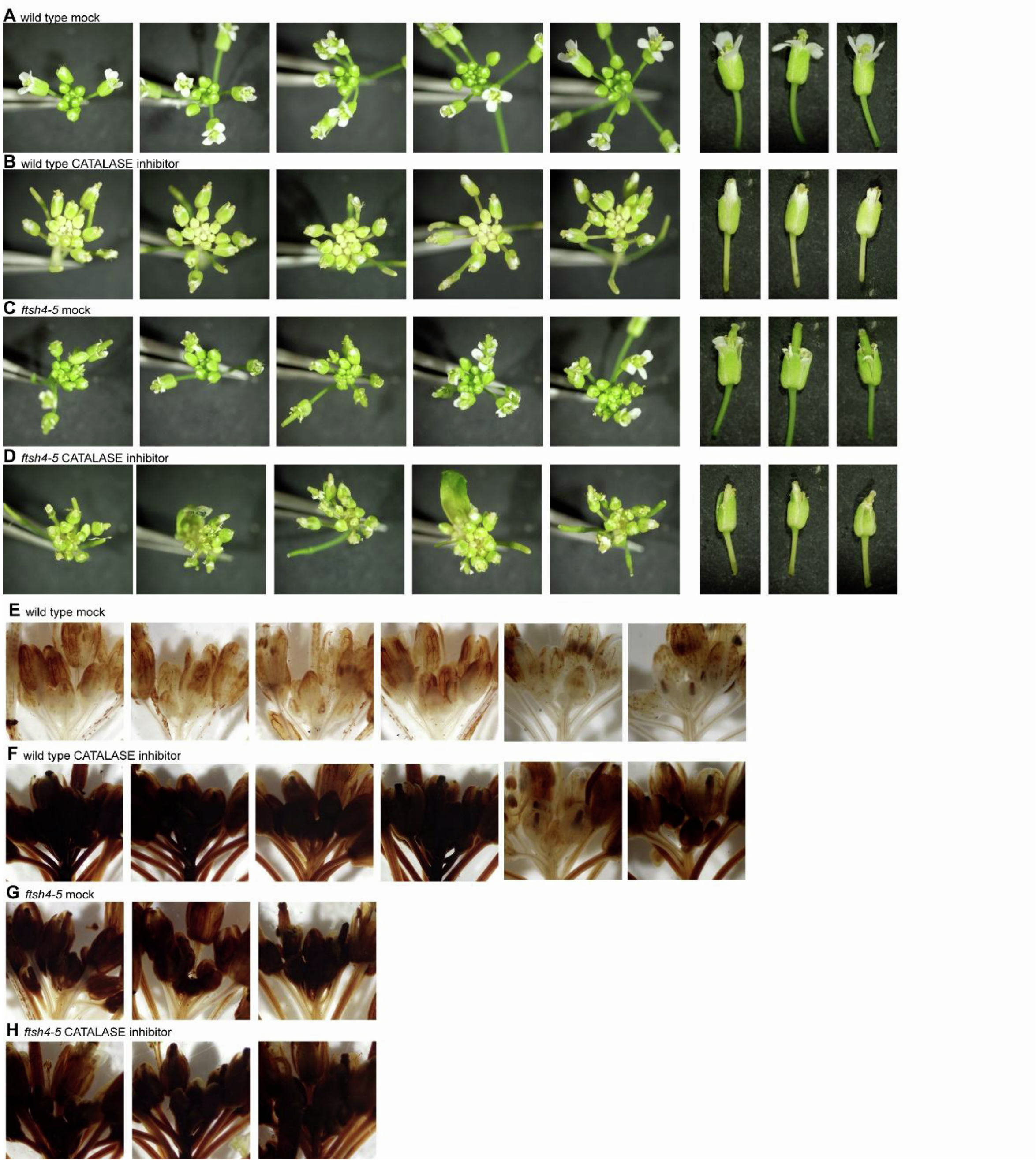


Figure S4: **Treatment with CATALSE inhibitor causes variable organ size and increases ROS, Related to Figure 4. A-D:** Pictures of inflorescences in wild type mock (A), wild type treated with CATALASE inhibitor (B), *ftsh4-5* mock (C), and *ftsh4-5* treated with CATALASE inhibitor (D). E-H Inflorescences are stained for hydrogen peroxide with DAB in wild type mock (E), wild type treated with CATALASE inhibitor (F), *ftsh4-5* mock (G), and *ftsh4-5* treated with CATALASE inhibitor (H).

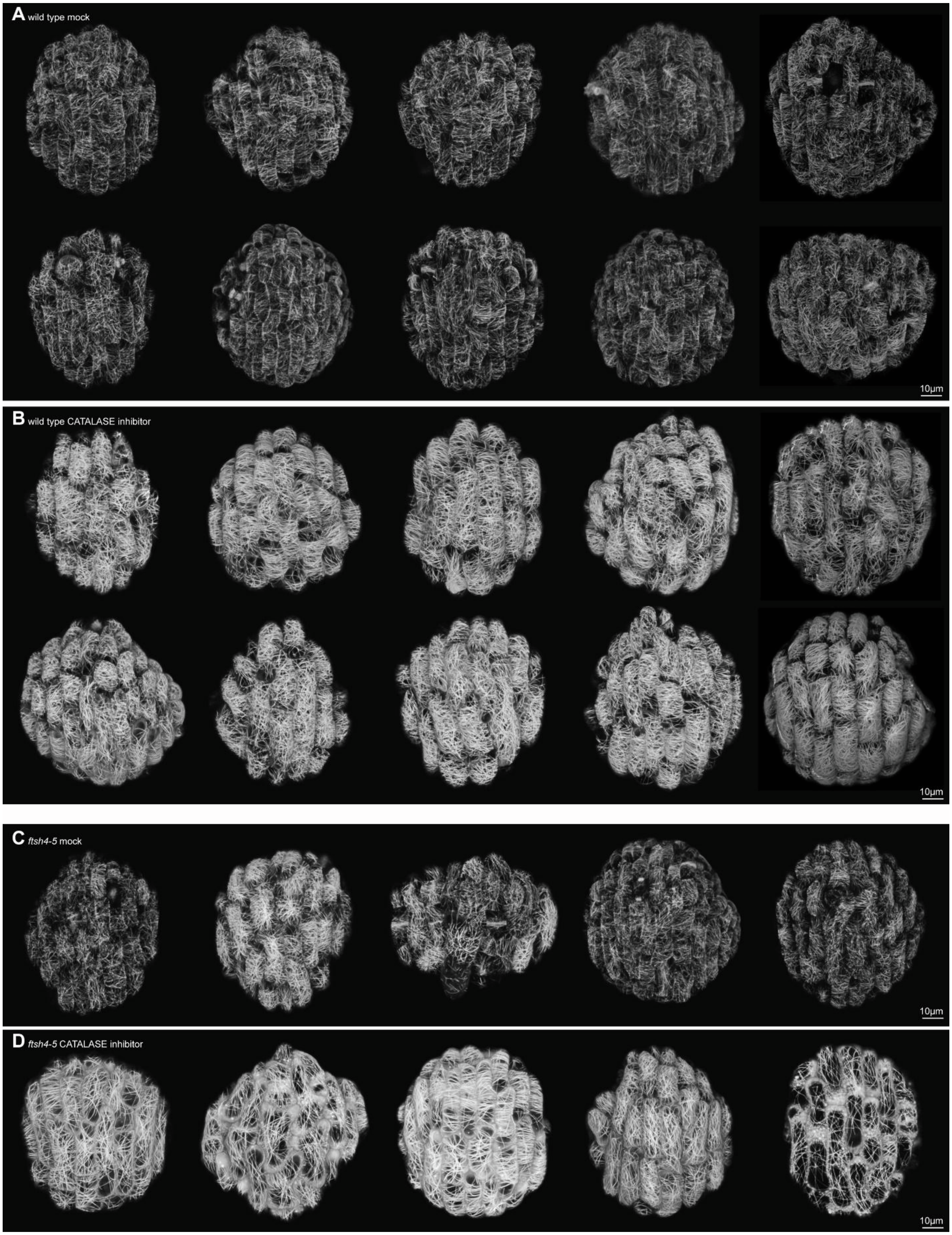


Figure S5: **ROS is sufficient to lead to crisscrossed microtubules, Related to Figure 4.** A-B:

Microtubules in (A) wild type mock-treated, (B) wild type CATALASE-inhibitor treated, (C) *ftsh4-5* mock treated, and (D) *ftsh4-5* CATALASE inhibitor treated (D) sepals. Other replicates are shown here.

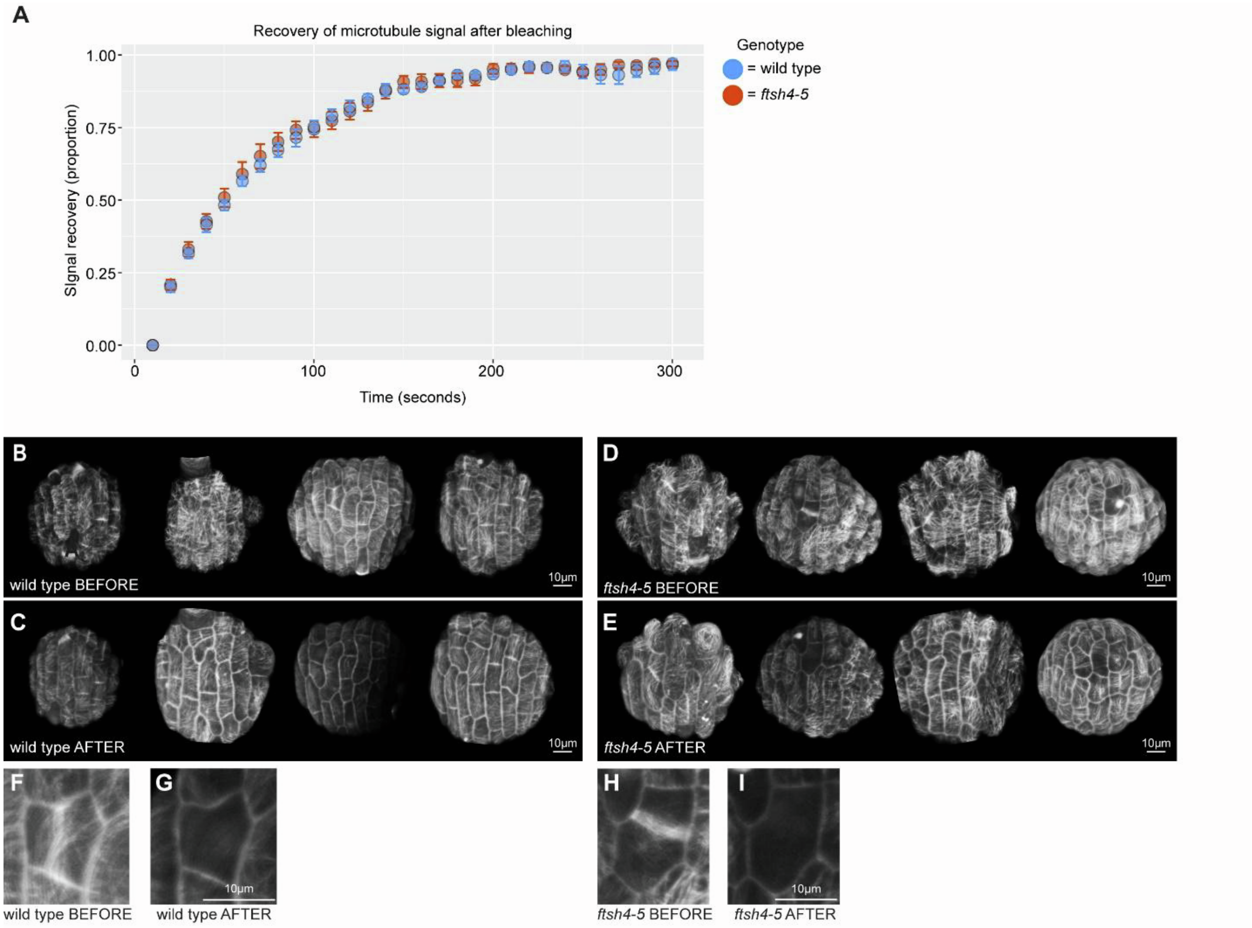


Figure S6: **Microtubules in *ftsh4-5* are more stable, Related to Figure 5.** A: Recovery of signal intensity of *p35S::RFP-TUB6*, which is indicative of rate of microtubule polymerization, after photobleaching in wild type and *ftsh4-5*. B-I: Other replicates of the propyzamide treatment to compare microtubule stability. (B) Wild type sepal cells before treatment and (C) the same wild type sepals after 30 min propyzamide treatment. (D) *ftsh4-5* sepal cells before treatment, and (E) the same *ftsh4-5* sepals after 30 min propyzamide treatment. F-I: Zoomed in to show that microtubules forming the preprophase band depolymerize in both wild type (F-G) and *ftsh4-5* (H-I) indicating that the treatment is effective in both genotypes.

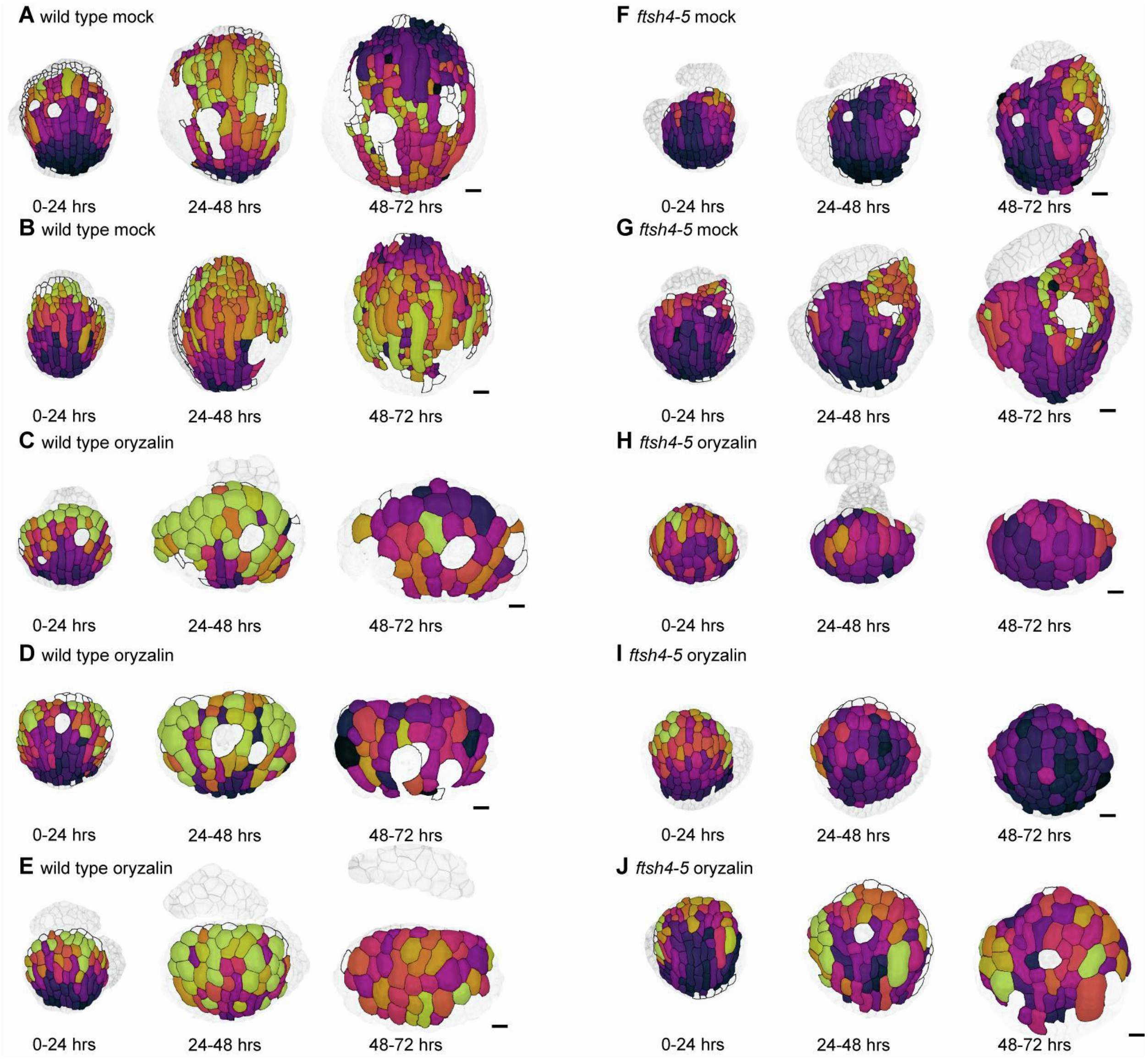


Figure S7: **Depolymerizing the microtubules is insufficient to restore growth heterogeneity, Related to Figure 6.** Cell area growth heat maps of sepal development with an oryzalin treatment. Sepals were imaged once every 24 hours for 4 days. Area growth is represented as a ratio and projected onto the later time point. The rest of the replicates are shown for (A-B) wild type mock treatment, (C-E) wild type oryzalin treatment, (F-G) *ftsh4-5* mock treatment, (H-J) *ftsh4-5* oryzalin treatment. Scale bars are 20 µm.

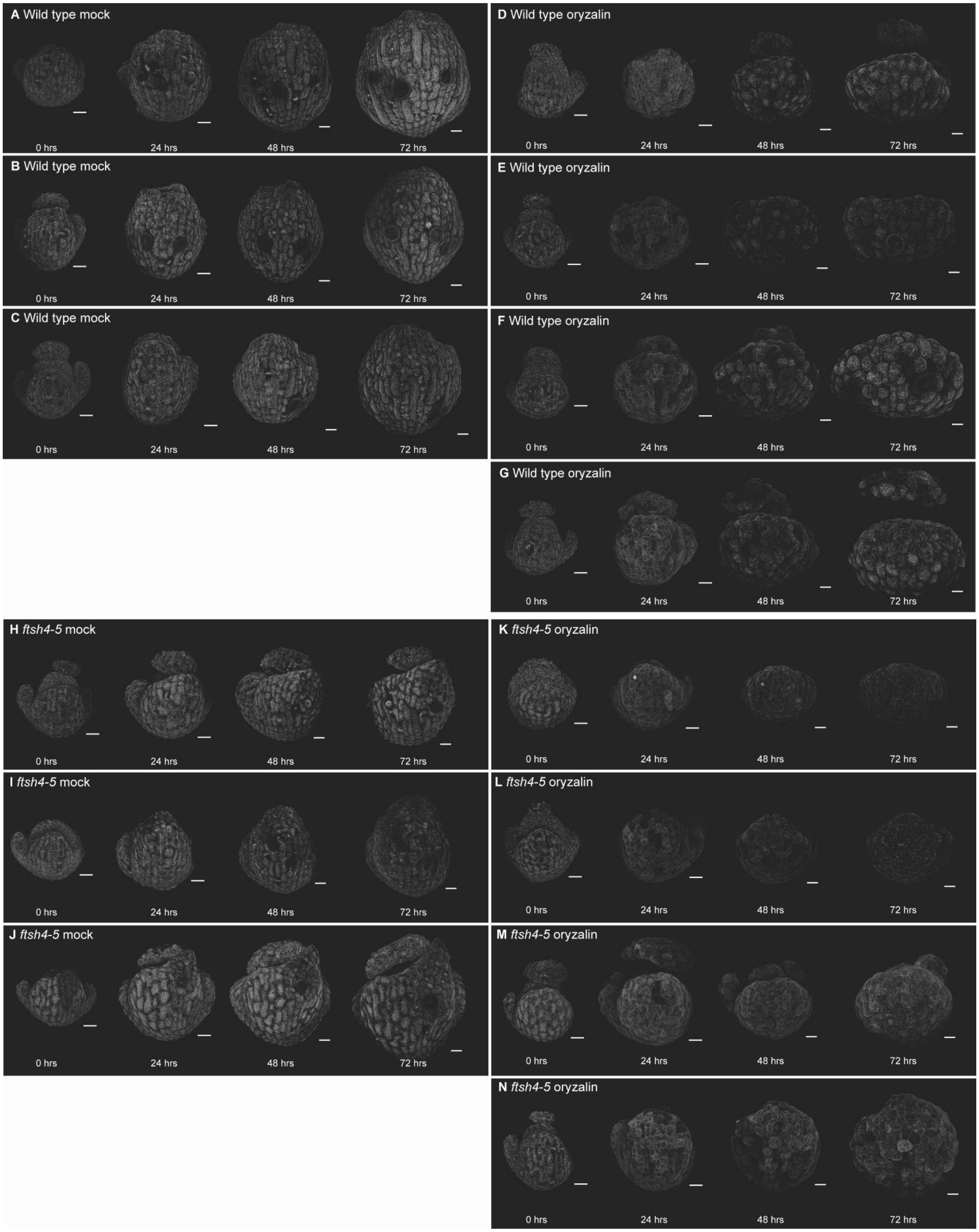


Figure S8: **Oryzalin treatment is effective in depolymerizing microtubules, Related to Figure 6.** Microtubule signal from the live imaging with the oryzalin treatment. All replicates are shown for wild type mock (A-C), wild type oryzalin (D-G), *ftsh4-5* mock (H-J), *ftsh4-5* oryzalin (K-N). Scale bars are 20 μm.
